# Hollow nanoreactors for Pd-catalyzed Suzuki–Miyaura coupling and *O*-propargyl cleavage reactions in bio-relevant aqueous media†
†Electronic supplementary information (ESI) available. See DOI: 10.1039/c8sc04390f


**DOI:** 10.1039/c8sc04390f

**Published:** 2018-12-26

**Authors:** Paolo Destito, Ana Sousa-Castillo, José R. Couceiro, Fernando López, Miguel A. Correa-Duarte, José L. Mascareñas

**Affiliations:** a Centro Singular de Investigación en Química Biolóxica e Materiais Moleculares (CiQUS) , Departamento de Química Orgánica , Universidad de Santiago de Compostela , 15782 , Santiago de Compostela , Spain . Email: joseluis.mascarenas@usc.es; b Department of Physical Chemistry , Center for Biomedical Research (CINBIO) , Southern Galicia Institute of Health Research (IISGS) , Biomedical Research Networking Center for Mental Health (CIBERSAM) , Universidade de Vigo , 36310 Vigo , Spain . Email: macorrea@uvigo.es; c Instituto de Química Orgánica General CSIC , Juan de la Cierva 3 , 28006 , Madrid , Spain . Email: fernando.lopez@csic.es

## Abstract

We describe the fabrication of hollow microspheres consisting of mesoporous silica nanoshells decorated with an inner layer of palladium nanoparticles and their use as Pd-nanoreactors in aqueous media. These palladium-equipped capsules can be used to promote the uncaging of propargyl-protected phenols, as well as Suzuki–Miyaura cross-coupling, in water and at physiologically compatible temperatures. Importantly, the depropargylation reaction can be accomplished in a bioorthogonal manner in the presence of relatively high concentrations of biomolecular components and even in the presence of mammalian cells.

## Abstract 2

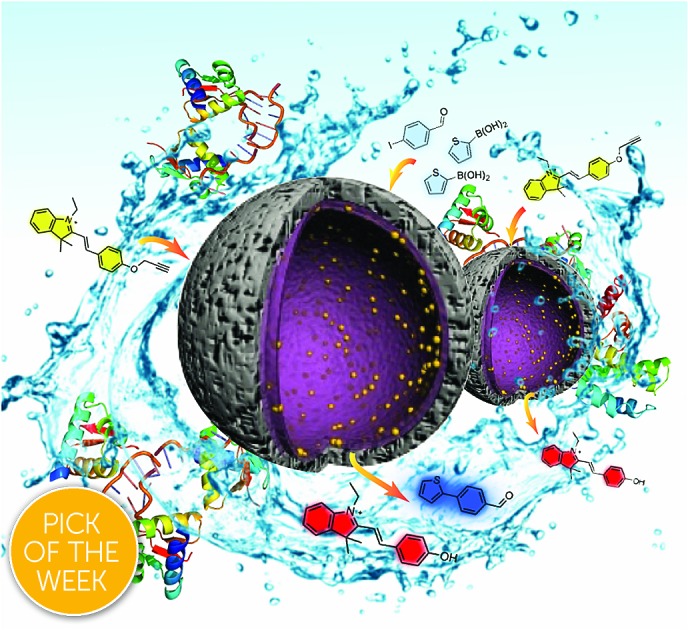
We describe the fabrication of hollow microspheres consisting of mesoporous silica nanoshells decorated with an inner layer of palladium nanoparticles and their use as Pd-nanoreactors in aqueous media.

## Introduction

During the last decade, bioorthogonal reactions have emerged as prominent tools in chemical biology and have facilitated the study and rational modification of different biological processes.^1^–^3^ Among these reactions, Cu-catalyzed azide–alkyne cycloaddition (CuAAC) remains as a main reference in the field.^4^–^7^ However, the coordination and oxidation lability of Cu(i) complexes and the toxicity associated with this metal have prompted the development of alternative, metal-free reactions (*e.g.*, strain promoted azide–alkyne cycloaddition, inverse electron demand Diels–Alder reactions, photoclick cycloaddition, among others).^8^–^17^

While these transformations represent important tools in chemical biology, bioorthogonal reactions catalyzed by metals are especially attractive owing to the additional control possibilities offered by the presence of the catalyst. However, achieving such metal-mediated processes in aqueous biological environments is far from obvious. Indeed, only recently several bioorthogonal metal-promoted reactions that take place in aqueous media, and even in cellular settings, have been developed.^18^–^24^ Among the different metals that have been explored, palladium occupies a prominent position owing to its well-known transformative potential in organic solvents.^18^–^27^ Although palladium complexes can be deactivated in aqueous solvents, Davis and Lin have independently demonstrated that in the presence of specific additives it is possible to achieve Pd-mediated protein Suzuki–Miyaura and Sonogashira cross-coupling reactions in an aqueous milieu.^28^–^32^ Chen has reported the use of Pd salts, including [Pd(allyl)Cl]_2_, to promote depropargylation and deallenylation reactions in proteins containing caged lysines and tyrosines.^33^–^35^ However, recent studies suggest that achieving these deallylation or depropargylation reactions in complex aqueous media, such as standard tissue cultures (HBSS and MEM), requires the use of more elaborated, specifically designed phosphine–palladium complexes.^36^–^38^

An alternative to small-sized, well-defined palladium reagents is the use of Pd nanoparticles (Pd-NPs). Indeed, a number of Pd-NP-promoted processes, including Suzuki–Miyaura cross coupling, have been achieved in aqueous media. However, most of them require the addition of bases and temperatures higher than 50 °C, which represent significant limitations in terms of translating this type of reactivity to biological media.^39^–^45^ Moreover, and importantly, catalytic Pd-NPs tend to aggregate and/or can be readily passivated when used in aqueous mixtures containing biological components. Chen and coworkers have shown that Pd-NPs can promote the uncaging of propargyloxy protected amines in cell-surface glycans;^46^ however they indicated that the nanostructures need to be immediately used after their generation (within 30 s). Unciti-Broceta and Bradley have devised an elegant method to protect the NPs from the media, by embedding them within polystyrene microspheres, which allowed their use in different biological set-ups.^47^–^51^ While these constructs have found interesting applications, the difficulties in controlling the architecture of the resulting structures, especially with regard to the distribution of Pd-NPs, might represent a limitation in terms of obtaining improved designs.

**Fig. 1:**
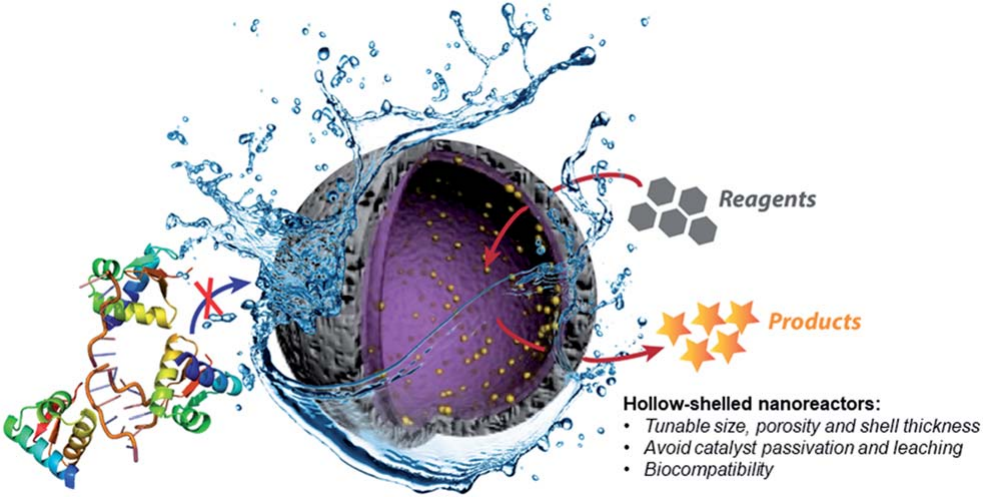
Prototype reactive chambers for metal-catalyzed biocompatible processes.

Therefore, the development of alternative palladium-based nanocatalytic constructions that work in biological media represents a major current challenge. In this context, we envisioned a new strategy to achieve bioorthogonal palladium heterogeneous catalysis based on the use of hollow nanoreactors. Specifically, we reasoned that porous hollow microspheres equipped with palladium nanoparticles at the internal surface might work as biocompatible, smart reactive chambers.^52^–^54^ The thickness and porosity of the chamber shell could be rationally tuned to allow an effective flow of low molecular weight reactants and products, while avoiding both metal leakage and the entrance of large biomolecules that could passivate the catalytic surface (Fig. 1).

While there are some precedents for the use of yolk@shell nanoparticles to carry out Pd-promoted processes (Suzuki–Miyaura coupling), the reactions have been performed in alcoholic rather than aqueous solvents, at non-physiological temperatures, and required high concentrations of the reactants.^55^–^59^

Herein we demonstrate that hollow microspheres consisting of mesoporous silica nanoshells equipped with Pd-NPs at their inner side behave as efficient Pd-nanoreactors for *O*-depropargylation reactions and Suzuki–Miyaura cross-couplings in aqueous buffer solutions (pH = 7.2). Importantly, while discrete palladium complexes like [Pd(allyl)Cl]_2_ failed to promote the propargyl-uncaging process when proteins like BSA are added to the reaction mixture (*vide infra*), our hollow nanoreactors remain active. Indeed, the capsules also work in cell culture medium and even in the presence of living cells.

## Results and discussion

**Scheme 1:**
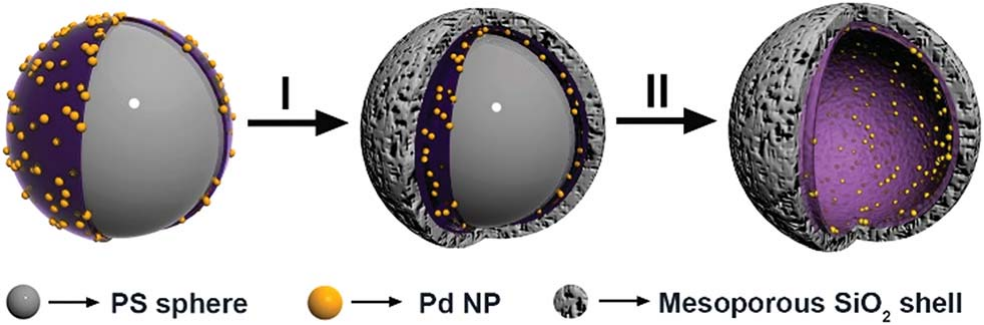
Strategy for the synthesis of hollow spheres equipped with Pd-NPs at their inner cavity.

The fabrication of the hollow-structured mesoporous systems equipped with palladium nanostructures at their inner cavity was achieved according to a protocol that ensures the tuning of the silica shell (Scheme 1).^56^,^60^–^62^ Namely, homogeneous polystyrene beads used as sacrificial templates are coated with polyallylamine hydrochloride (PAH). Then, freshly prepared Pd nanoparticles (3.8 ± 0.5 nm, Fig. S1†) are deposited onto these polystyrene particles and subsequently coated with a homogeneous mesoporous silica layer using a tetraethoxysilane solution in ethanol (5%). The use of a polystyrene core facilitated its removal under soft conditions (3 : 1 THF/H_2_O), avoiding alternative calcination processes that could sinter and reduce the catalytic potential of the Pd-NPs (see the ESI for details, Fig. S2†).

**Fig. 2:**
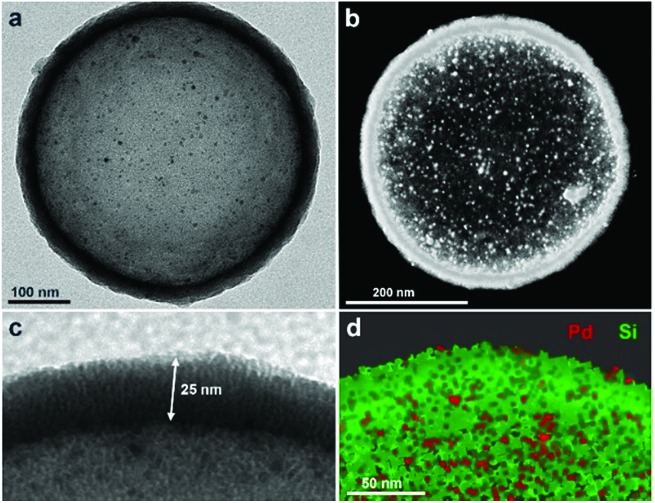
Characterization of **Pd-Cap1**. (a) Representative TEM image (500 nm diameter); (b) STEM image; (c) zoom-in view showing the vertically oriented mesopores in the silica shell with a thickness of 25 nm; (d) XEDS mapping showing the elemental distribution of Pd and Si.

Fig. 2 shows typical transmission electron microscopy (TEM) images of the resulting nanocapsules exemplified by **Pd-Cap1** with a homogeneous average size of 500 nm and a mesoporous silica shell thickness of 25 nm.^60^–^62^ The Pd-NPs are homogeneously distributed through the inner surface of this mesoporous shell as confirmed by STEM and EDS analyses (Fig. 2b and d and S3†). The content of Pd in these capsules, measured by ICP-MS, turned out to be 0.61 μg mg^–1^.

**Scheme 2:**
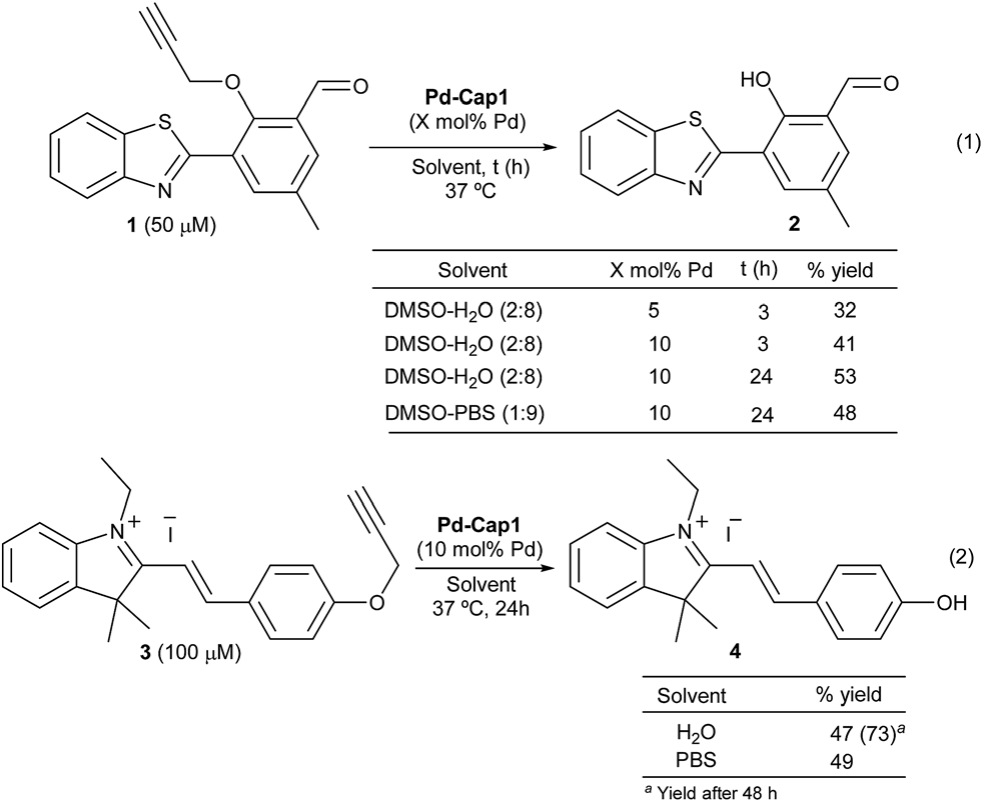
**Pd-Cap1**-promoted uncaging of **1** and **3**. Conditions: a suspension of **Pd-Cap1** (0.2 mL, 8.33 mg mL^–1^) in EtOH was centrifuged (8000 rpm) for 10 min. The supernatant was removed and a mixture of H_2_O : DMSO (4 : 1) was added, followed by addition of **1** or **3**. The mixture was stirred at 37 °C, under air, for the indicated time and centrifuged (8000 rpm) prior to quantification by fluorescence analysis (for **2**) or by HPLC (for **4**).

Prior to the reactivity tests, we checked the stability of the Pd nanocapsules (**Pd-Cap1**) in water at 37 °C. Gratifyingly, the structural features of the spheres and the size of the internalized Pd-NPs remained unaffected even after 30 days, confirming the protective role of the mesoporous shells in the characteristics of the Pd-NPs. The catalytic activity was preliminarily tested in the uncaging of propargyl phenol derivative **1**, a pro-fluorogenic substrate which upon deprotection provides a fluorescent benzothiazole (**2**, Scheme 2, eqn (1)). The green fluorescence emission of this product (*λ*_exc_ = 460 nm and *λ*_em_ = 535 nm), which arises from an excited state intramolecular proton transfer (ESIPT) process, enables an efficient quantification of reaction yields at low μM concentrations.^63^ Treatment of **1** (50 μM) with **Pd-Cap1** (5 mol% Pd content) in a 2 : 8 mixture of DMSO : water provided, after 3 h at 37 °C, the desired product (**2**) in 32% yield (the remaining starting material (**1**) accounts for the rest of the mass balance). Control experiments confirmed that the reaction does not proceed in the absence of **Pd-Cap1**.^64^ The yield could be improved to 53% by doubling the concentration of **Pd-Cap1** (10 mol% of Pd content) and running the reaction for 24 h. Importantly, control experiments with non-encapsulated Pd-NPs, stabilized with polyvinylpyrrolidone (PVP, **Pd-NP1**), under otherwise identical reaction conditions, provided yields below 10% after 24 h, confirming that confinement of the Pd-NPs in the nanoreactor has a pivotal influence on their catalytic activity.^64^

Remarkably, the reaction promoted by **Pd-Cap1** could also be carried out in PBS, instead of water, with similar results (48% yield). To avoid the use of DMSO as the co-solvent, we checked the reactivity of the styryl indolinium probe **3** (Scheme 2, eqn (2)), which presents excellent water solubility in the μM range. Interestingly, the reaction outcome can be easily judged by naked-eye detection of the colorimetric change, and yields are easily quantified by HPLC-MS.^64^ Treatment of a water solution of **3** with **Pd-Cap1** (10 mol% of Pd) provided, after 24 h at 37 °C, the depropargylated product **4** in 47% yield, while a significantly better value of 73% could be obtained if the reaction was carried out for 48 h (Scheme 2, eqn (2)). A similar yield of **4** after 24 h was obtained when the reaction was carried out in PBS (Scheme 2, eqn (2)). Overall, despite the yields and reaction rates are moderate, these results validate the potential of the palladium nanoreactors in water, at low temperatures and under high dilution conditions.

**Scheme 3:**
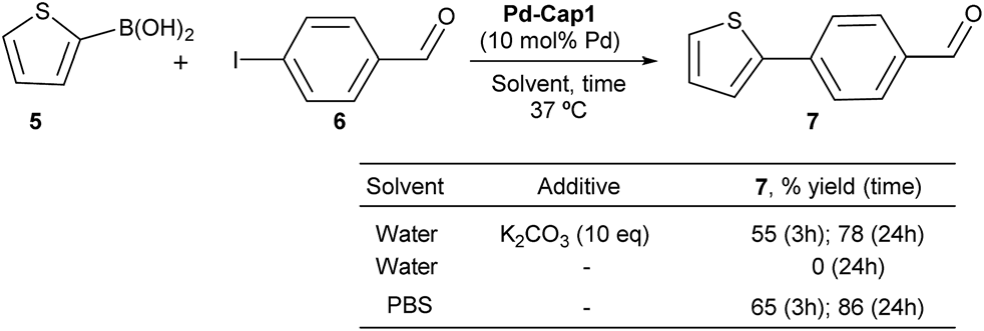
Suzuki–Miyaura cross-couplings of **5** and **6**.

To check whether these Pd-nanoreactors could be applied to more challenging bimolecular processes, we tested their effectivity in Suzuki–Miyaura coupling between 2-thiophenyl boronic acid (**5**) and *p*-iodo benzaldehyde (**6**) (Scheme 3). The expected product, 4-(thiophen-2-yl)benzaldehyde (**7**), shows a strong blue fluorescence (*λ*_exc_ = 330 nm, *λ*_em_ = 434 nm, 10 μM in EtOH : H_2_O 1 : 1),^65^ facilitating reaction monitoring. Gratifyingly, when a water suspension of **Pd-Cap1** (10 mol% Pd content), **5** (150 μM), **6** (100 μM) and K_2_CO_3_ (1 mM) was mixed at 37 °C for 3 h, the desired product **7** was obtained in 55% yield. Moreover, by running the reaction for 24 h, the yield of **7** could be further increased up to 78%. Not surprisingly, in the absence of a base the reaction did not occur. However, we were pleased to find that the reaction could be carried out using PBS 10× (pH 7.2) as solvent, without any additional base. In this case, a 65% yield of **7** was obtained after 3 h at 37 °C, while after 24 h the yield was 86%. *Notably, this reaction represents the first application of hollow palladium nanocapsules in Suzuki–Miyaura cross coupling in an aqueous solvent and at physiological temperatures*.

Although it was not the main goal of this research, it is worth noting that these nanoreactors can be easily recovered by centrifugation after reaction completion, and preliminary experiments demonstrate that they can be reused, at least three times, without an important loss of catalytic activity (Fig. S16†).^64^ On the other hand, leaching was insignificant, as determined by ICP-MS analysis of the supernatants. Accordingly, mother liquids obtained by centrifugation after reaction did not show catalytic activity.^64^

**Scheme 4:**
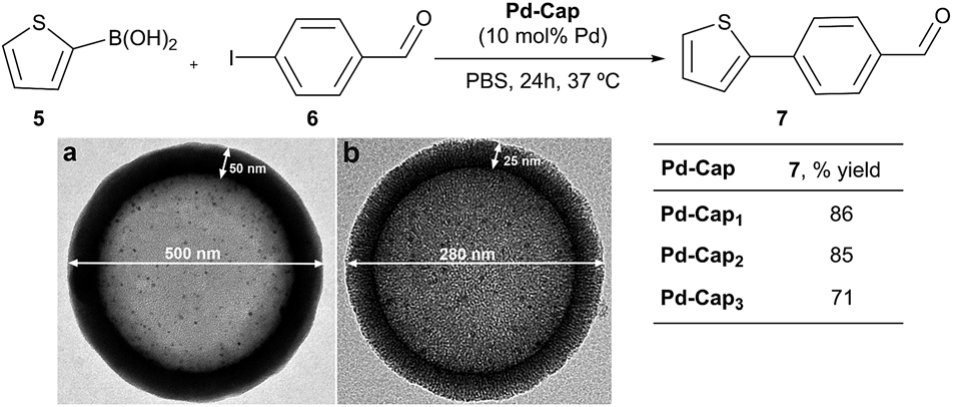
Reactivity of Pd-capsules with different diameters and thicknesses. (a) TEM image of **Pd-Cap3**; (b) TEM image of **Pd-Cap2**.

We next analyzed the influence of the structural properties of the nanostructures on the reactivity. Thus, two additional Pd-nanocapsules of different sizes and shell thicknesses were prepared.^64^ Capsules **Pd-Cap2**, which are significantly smaller (280 nm) but of equal shell thickness (25 nm), were prepared from polystyrene beads of 230 nm (see Scheme 4 for the TEM image). On the other hand, **Pd-Cap3**, comprising a thicker mesoporous shell (50 nm), with an overall diameter of 500 nm were obtained following a slightly modified procedure.^64^ As can be deduced from the results of Scheme 4, **Pd-Cap2** showed a very similar behavior in the cross-coupling between **5** and **6**, providing an 85% yield of **7** after 24 h at 37 °C. On the other hand, the efficiency of **Pd-Cap3**, which features a 50 nm shell width, turned out to be slightly lower (**7**, 71% yield), indicating that increasing the shell thickness disrupts the catalytic activity, likely because of a less efficient flow of reactants and products throughout the shell. These results confirm that tuning the thickness of the surface allows extra control over the reaction kinetics.

**Fig. 3:**
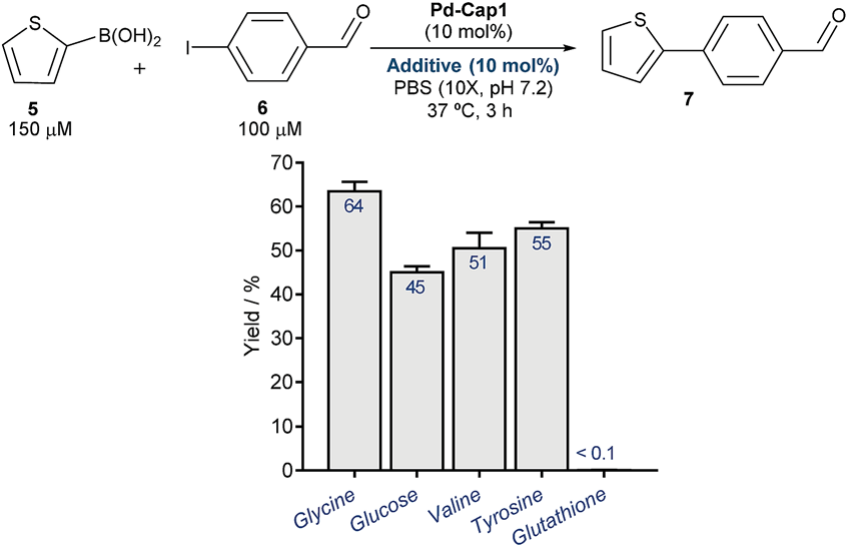
**Pd-Cap1**-promoted Suzuki–Miyaura reaction of **5** and **6** (top, mean ± s.e.m., *n* = 2), in the presence of additives.

At this point, we explored the viability of translating the above reactivities to aqueous media containing additional biomolecules, in order to test the bioorthogonality of the approach. Gratifyingly, the Suzuki–Miyaura coupling between **5** and **6**, promoted by **Pd-Cap1**, proceeds with similar yields in the presence of one equivalent (with respect to the Pd content) of carbohydrates, such as glucose, or different amino acids like glycine, tyrosine or valine (Fig. 3). We later found that it also tolerates larger excesses of the additives (Fig. S11†). However, the reaction was inhibited by biorelevant thiols such as glutathione and did not proceed in cell lysates or in the presence of Vero cells. This inactivation is likely associated with the mechanistic complexity of the bimolecular process and the lateral reactivity of transient palladium intermediates with some of the components of the mixture (especially with thiols).

**Fig. 4:**
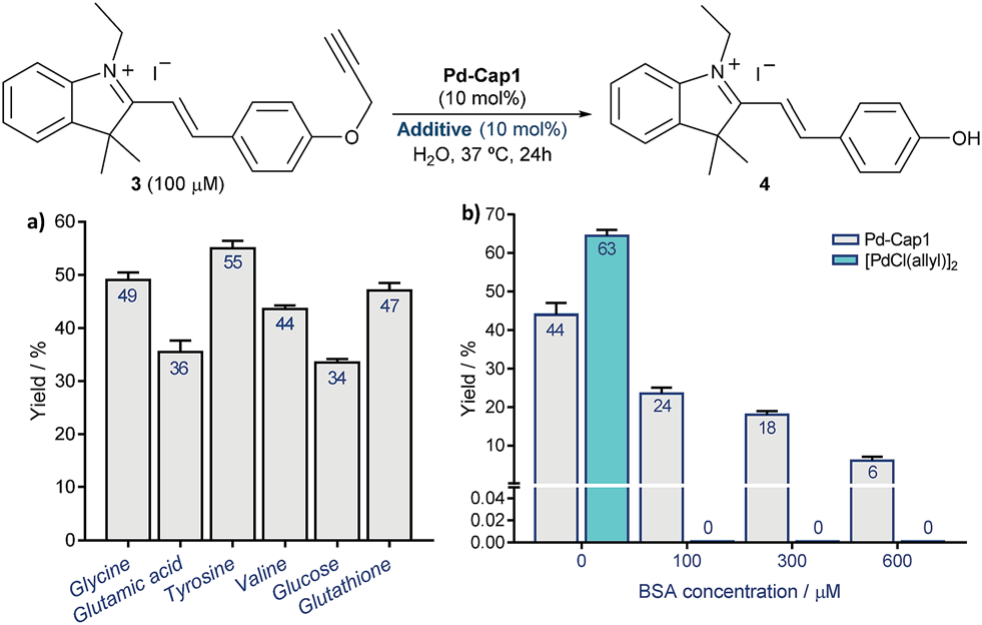
**Pd-Cap1**-promoted depropargylation of **3** (mean ± s.e.m., *n* = 2) in the presence of different additives, 1.0 equiv. with respect to Pd (a), and of increasing concentrations of BSA (b). The reaction is also effective in the presence of higher amounts of the additives as indicated in (a) (see Fig. S12).†

However, to our delight, *the depropargylation of probe***3***proceeded in the presence of added glucose and different types of amino acids and even in the presence of glutathione* (Fig. 4a and S12†). Interestingly, the reaction can also be performed at acidic pHs of 5.3 and 4.5 (45% and 46% yield, respectively). Moreover, the reaction is also effective in the presence of significant amounts of proteins like BSA (up to 600 μM, Fig. 4b). Importantly, under these conditions, discrete Pd complexes that had been previously used in lysine uncaging reactions, like [Pd(allyl)Cl]_2_,^33^–^35^ turned out to be completely ineffective, even at BSA concentrations as low as 100 μM. These results confirm the superiority of the Pd-nanocapsules, which prevents an otherwise rapid passivation.

Finally, a preliminary analysis of the toxicity in Vero cells revealed a superior biocompatibility of the nanoreactor **Pd-Cap1**, compared to [Pd(allyl)Cl]_2_. Indeed, while at low concentrations (2.5 μM), none of the Pd-systems were significantly toxic, at a concentration of 20 μM a significant toxicity of [Pd(allyl)Cl]_2_ was observed (85% of cells were dead after 24 h), whereas the Pd-nanocapsules still provided a good cell viability > 75% (Fig. S14†).^64^

Given their low toxicity, we studied their catalytic activity in cellular environments. With this purpose, we performed the depropargylation of the probe HBTPQ (**8**)^66^ in the presence of Vero mammalian cells.

**Fig. 5:**
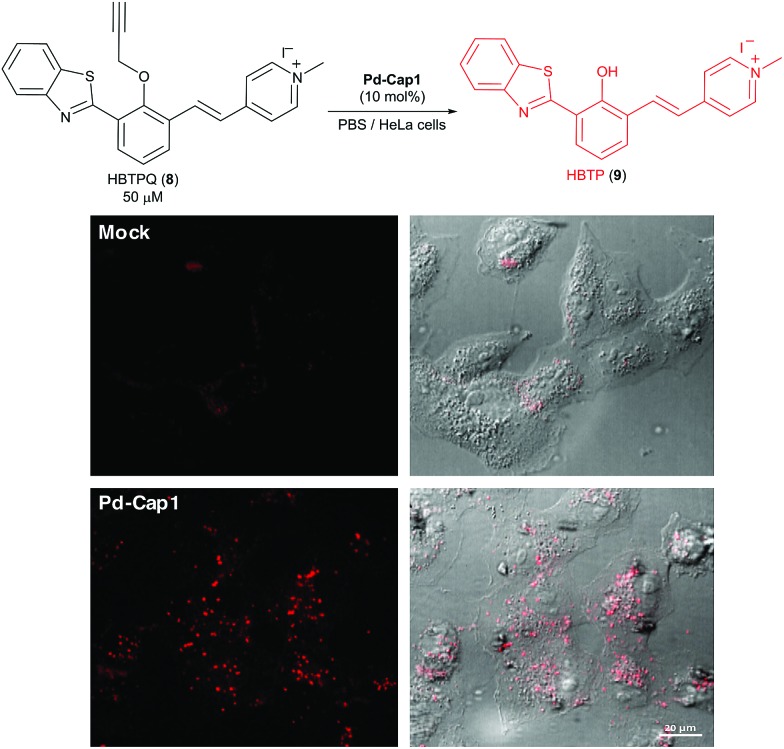
Micrographs of Vero cells incubated for 30 min in DMEM containing **Pd-Cap1** (≈5 μM Pd) and 50 μM HBTPQ (**8**), washed and analyzed.

This probe does not emit red fluorescence when irradiated with UV light but the cleavage of its propargyl moiety generates the red fluorescent compound HBTP (**9**, *λ*_exc_ = 330 nm, *λ*_em_ = 635 nm). In this experiment, the extracellular medium was removed and substituted with a solution of 50 μM HBTPQ (**8**) in DMEM or in PBS. **Pd-Cap1** was added to reach a final 5 μM concentration of Pd, and the resulting cellular fluorescence was monitored by confocal microscopy. Gratifyingly, red fluorescence spots arising from the intracellular presence of the product were observed in the cells incubated with probe **8** (Fig. 5).

## Conclusions

To sum up, we have demonstrated that hollow microspheres consisting of mesoporous silica nanoshells with Pd-NPs at their inner face behave as efficient nanoreactors for Pd-catalyzed depropargylation and Suzuki–Miyaura intermolecular cross-coupling under physiologically compatible aqueous conditions. The aqueous stability, monodispersity, well-defined architecture and fabrication reproducibility of the capsules represent important advantages compared to other polymer-based structures. Moreover, their low toxicities to cells owing to an inert external surface and the bioorthogonality of the depropargylation augur well for further developments with hollow nanoreactors that contain well-defined metal catalysts inside the capsule. The development of this type of biocompatible reactor might allow the implementation of new therapies based on prodrug activation^67^ and/or contribute to the establishment of biocompatible, non-natural metabolic networks. Work in this direction is underway.

## Conflicts of interest

There are no conflicts to declare.

## Supplementary Material

Supplementary information

Infographic
