# Supplementary material for: Hollow nanoreactors for Pd-catalyzed Suzuki–Miyaura coupling and O-propargyl cleavage reactions in bio-relevant aqueous media
Source: Chem Sci. 2018 Dec 26;10(9):2598–603. doi: 10.1039/c8sc04390f (PMC6419927; doi:10.1039/c8sc04390f)
Supplement: Supplementary file 1 [file SC-010-C8SC04390F-s001.pdf]

Electronic Supplementary Material (ESI) for Chemical Science.
This journal is © The Royal Society of Chemistry 2019

SUPPLEMENTARY INFORMATION

Hollow-Shelled Pd-Nanoreactors for Suzuki-Miyaura Couplings and O-Propargyl Cleavage Reactions in Bio-relevant Aqueous Media

Paolo Destito,[a,§] Ana Sousa,[b,§] José R. Couceiro,[a] Fernando López,*[,a,c] Miguel A. Correa-Duarte,*[,c] José L. Mascareñas*[,a]

[a] Centro Singular de Investigación en Química Biolóxica e Materiais Moleculares (CiQUS) and Departamento de Química Orgánica. Universidad de Santiago de Compostela, 15782, Santiago de Compostela, Spain

[b] Department of Physical Chemistry, Center for Biomedical Research (CINBIO), Southern Galicia Institute of Health Research (IISGS), and Biomedical Research Networking Center for Mental Health (CIBERSAM), Universidade de Vigo, 36310 Vigo, Spain

[c] Instituto de Química Orgánica General CSIC, Juan de la Cierva 3, 28006, Madrid, Spain

[§] These authors contributed equally to this work.

* Dr. Fernando López. E-mail: fernando.lopez@csic.es ; * Prof. José L. Mascareñas. E-mail: joseluis.mascarenas@usc.es ;* Prof. Miguel A. Correa. E-mail: macorrea@uvigo.es

TABLE OF CONTENTS

	General Procedures	S3
	Synthesis of Pd nanoreactors (**Pd-Cap1** – **Pd-Cap3**)	S5
	Procedure for the synthesis of substrates **3** and **4**	S7
	Procedure for the propargyl uncaging	S9
	General Procedure for the Suzuki-Miyaura cross coupling	S13
	Procedures for catalysis in the presence of bio-additives	S16
	Procedures for catalysis in cell cultures	S20
	Procedure to evaluate leaching from Pd-Capsules	S21
	Recyclability test of **Pd-Cap1**	S22
	References	S23
	NMR Spectra	S24

General Procedures

Tetraethylorthosilicate 98% (TEOS), ammonium hydroxide solution 28−30% (NH_4OH), poly(allylamine hydrochloride) (PAH, MW: 17500), sodium chloride (NaCl), cetyltrimethylammonium bromide (CTAB), Sodium tetrachloropalladate(II) (Na_2PdCl_4), polyvinylpyrrolidone (PVP, MW: 30,000), L-ascorbic acid (AA) were purchased from Sigma-Aldrich. PS beads were purchased from Ikerlat. Compounds **S3**, **5** and **6** are commercially available and have been purchased from Sigma-Aldrich. Compounds **S1**,[1] **S2**,[2] **1**,[3] **7**,[4] **HBTPQ** (**8**) and **HBTP** (**9**)[5] are known compounds and were synthesized according to those previously reported procedures. Reactions were conducted in dry solvents under nitrogen atmosphere unless otherwise stated. Dry solvents were freshly distilled under argon from an appropriate drying agent before use. The removal of solvents under reduced pressure was carried out on a rotary evaporator. Water was deionized and purified on a *Millipore Milli-Q Integral* system. The abbreviation "rt" refers to reactions carried out approximately at 23 °C. Reaction mixtures were stirred using Teflon-coated magnetic stirring bars. Reaction temperatures were maintained using Thermo watch-controlled silicone oil baths. Thin-layer chromatography (TLC) was performed on silica gel plates (*Merck* 60 silica gel F_{254}) and components were visualized by observation under UV light, and / or by treating the plates with *p*-Anisaldehyde followed by heating. Flash chromatography was carried out in silica gel (*Merck* Geduran Si 60, 40 – 63 µm silica gel, normal phase) unless otherwise stated. Dryings were performed with anhydrous Na_2SO_4 or $MgSO_4$. Concentration refers to the removal of volatile solvents via distillation using a Büchi rotary evaporator followed by residual solvent removal under high vacuum.

NMR spectra were recorded in $CDCl_3$ and CD_3OD at 300 MHz (Varian), 400 MHz (Varian) or 500 MHz (Bruker and Varian). Carbon types and structure assignments were determined from DEPT-NMR and two-dimensional experiments (HMQC and HMBC and COSY). NMR spectra were analyzed using MestreNova© NMR data processing software (www.mestrelab.com). The following abbreviations are used to indicate signal multiplicity: s, singlet; d, doublet; t, triplet; q, quartet; p, pentuplet; dd, double doublet; m, multiplet; br, broad.

Mass spectra were acquired using IT-MS Bruker AmaZon SL at CIQUS and also using electrospray ionization (ESI) and were recorded at the CACTUS facility of the University of Santiago de Compostela. UV and fluorescence spectra were acquired using Jasco V-670 spectrometer and Varian Cary Eclipse fluorescence spectrofluorometer. LC-MS analysis was carried out using Bruker Amazon IT/MS with C18 column.

Confocal images were acquired with a Andor DragonFly spinning disc confocal microscope.

Electron microscopy: TEM images were obtained using a JEOL JEM 1010 transmission electron microscope operating at an acceleration voltage of 100 kV. HRTEM and elemental mapping by XEDS analysis were carried out with a JEOL JEM 2010F transmission electron

microscope operating at an acceleration voltage of 200 kV.
Energy-dispersive X-ray spectroscopy: XEDS experiments were performed on a JEOL JSM-6700F high resolution transmission electron microscope.
UV-visible spectroscopy: UV−Vis absorbance spectra were recorded on a HP8453 UV−Vis spectrophotometer fitted with a thermostated holder and collected from a 1-cm pathlength quartz cuvette.
ICP-AES The Pd content of the samples was determined by an inductively coupled plasma atomic emission spectrometer (ICP-AES, POLY SCAN 60 E).

Synthesis of Pd nanoreactors (Pd-Cap1 – PdCap3)

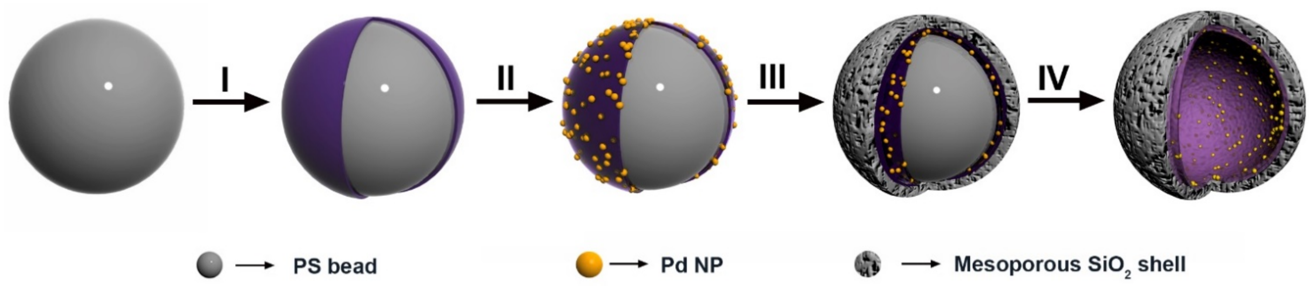

I) Functionalization of PS beads. 500 nm PS beads were functionalized with PAH. To this end, PAH was dissolved in 0.5 M NaCl (pH 5.0) with a final polymer concentration of 1 mg mL^{-1}. Then, 25 mL of the positively charged PAH solution were added to the PS beads (12.5 mg) and stirred at room temperature for 30 min. The excess of reagents was removed by four centrifugation-redispersion cycles with water (9000 rpm, 40 min). This polyelectrolyte film provides the PS particles with the necessary electrostatic charge for the later adsorption of Pd-NPs.

II) Synthesis of 3.8 nm Pd NPs and their deposition on functionalized PS beads. Pd NPs were synthesized according to a method described in the literature with slight modifications.[6] In a typical procedure, an aqueous solution (22.5 mL) containing PVP (262.5 g) and AA (18.75 mg) was heated at 100 °C under reflux for 10 min. Subsequently, an aqueous solution (5.0 mL) containing Na_2PdCl_4 (10 mM) was added. The reaction was allowed to continue at 100 °C for 3 h to obtain Pd-NPs that were subsequently separated from the reaction mixture by centrifugation (17500 rpm, 90 h). The collected Pd NPs (**Pd-NP1**) were washed several times with water to remove excess PVP. Finally, they were redispersed in water (20 mL). Size distribution analysis of these Pd nanoparticles (Pd-NPs) showed a diameter of 3.81nm ± 0.5 (see Figure S1)

A solution of 3.8 nm Pd-NPs (10 ml) was added to 50 mL of functionalized PS beads (1 mg mL^{-1}). The mixture was stirred at room temperature for 30 min. The excess of Pd-NPs was removed by three centrifugation redispersion cycles with water (4500 rpm, 40 min). Finally, the product was redispersed in water (10 mL) (see Figure S2a)

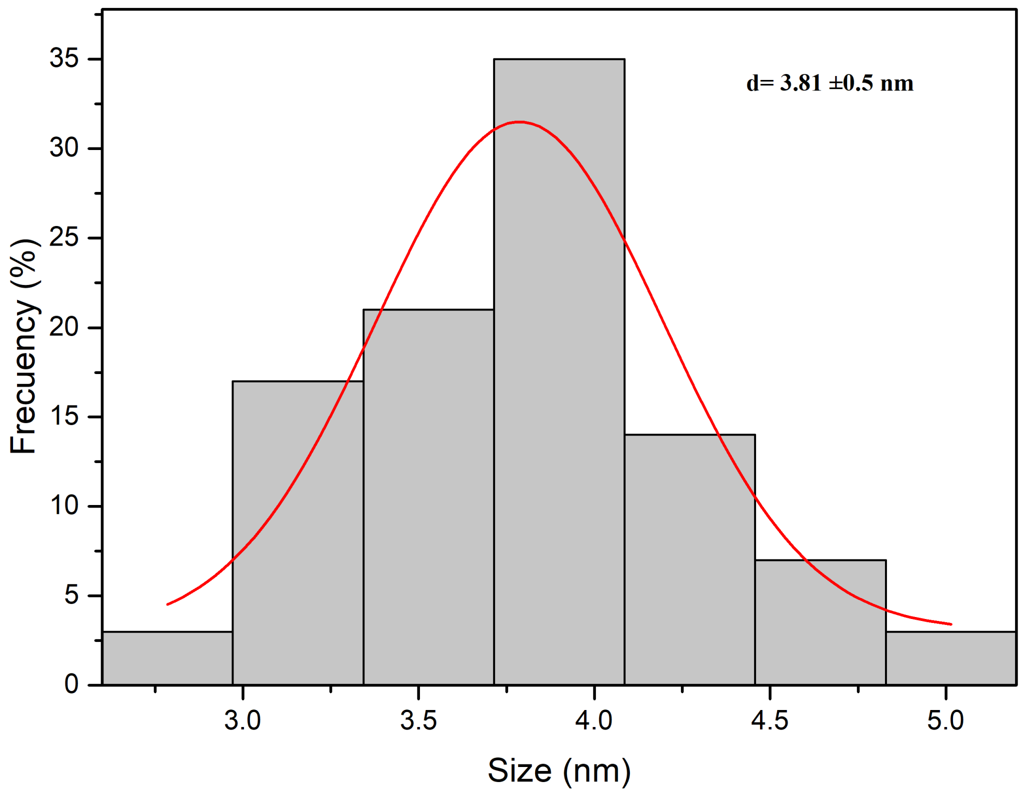

Figure S1. Size distribution analysis of the Pd nanoparticles (Pd-NPs) used.

III-IV) Mesoporous silica coating and preparation of the hollow nanoreactor Pd-Cap1. Different concentrations of a PS/Pd-NPs suspension (5 mg mL^{-1}) were added drop by drop under sonication to a solution composed by CTAB (100 mg), deionized water (35 mL), ethanol (35 mL) and ammonia aqueous solution (28 wt%, 0.365 mL). The resulting suspension was homogenized by sonication for 15 min. Then, 1 mL of a 5 % (v/v) TEOS solution in EtOH was added dropwise to the previous suspension under sonication. This mixture was stirred for 24 h in order to obtain a 25 nm homogeneous silica growth. The resulting composite was washed by four centrifugation/redispersion cycles with EtOH (see Figure S2b).

Finally, PS and CTAB templates were removed with a THF/H_2O solution under sonication and the Pd/SiO_2 mesoporous capsules were redispersed in EtOH (10 mL) (see Figure S2c).

NOTE: For the synthesis of **Pd-Cap2**, polystyrene beads of 230nm were employed in the step **I**. For the synthesis of **Pd-Cap3** (shell thickness of 50nM), a PS/Pd-NPs suspension of 2.5 mg mL^{-1} was used in step **III**.

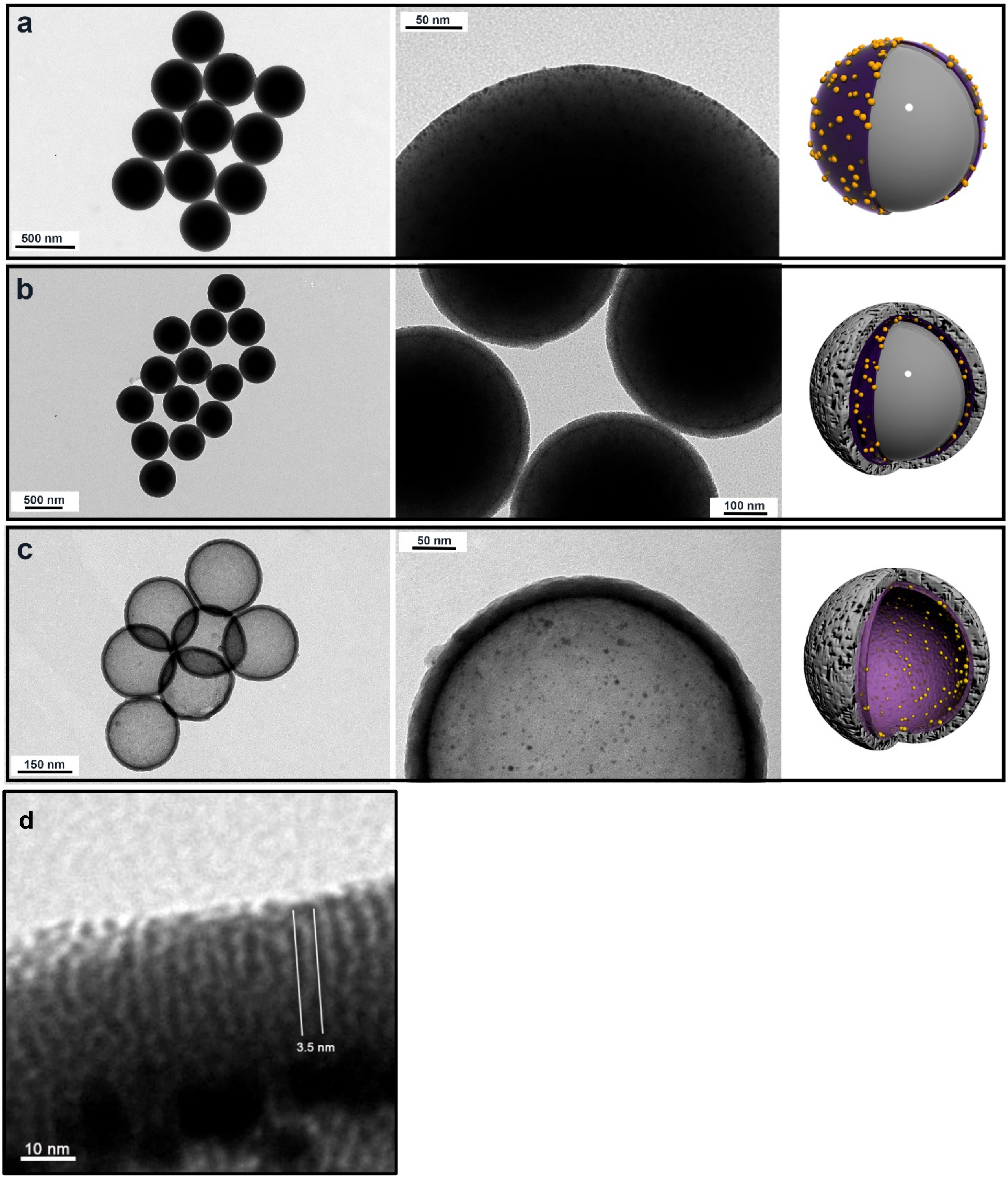

Figure S2. TEM images of the different synthetic steps during the fabrication of the nanoreactors. a) PS beads covered by PAH and desposited Pd-Nps [PS/Pd-Nps]; b) Mesoporous silica coated particles; c) hollow naoreactor **Pd-Cap1;** d) vertically oriented mesoporous channels (3.5 nm) present in the silica shell

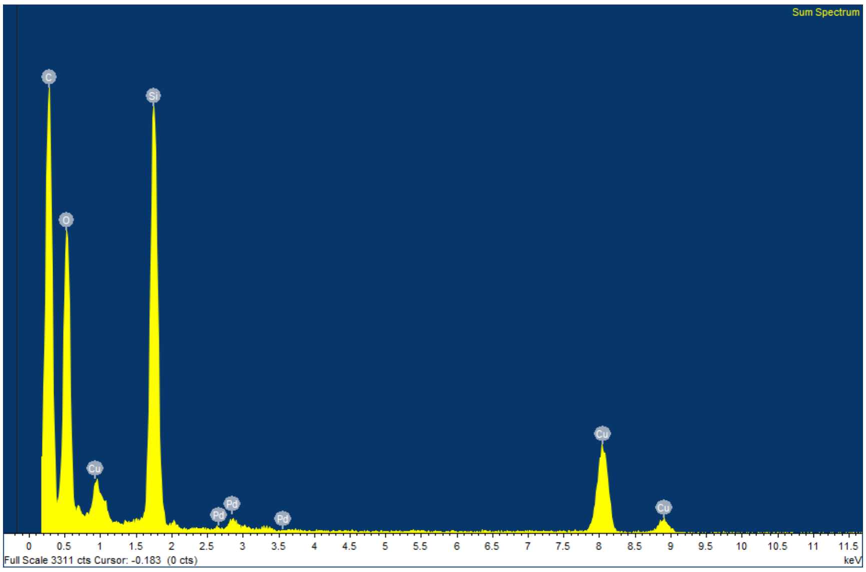

Figure S3. EDS analysis of the **Pd-Cap1** showing the presence of Pd and Si

Procedure for the synthesis of substrates 3 and 4 (exemplified for the synthesis of **3**)

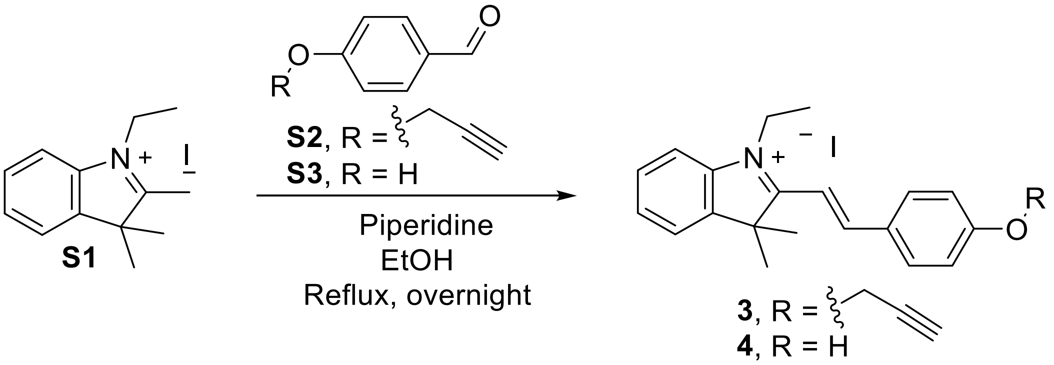

(E)-1-ethyl-3,3-dimethyl-2-(4-(prop-2-yn-1-yloxy)styryl)-3H-indol-1-ium iodide (**3**):

1-Ethyl-2,3,3-trimethyl-3*H*-indol-1-ium iodide (**S1,** 100 mg, 0.317 mmol, 1eq.) was dissolved in ethanol (3 mL), and piperidine (0.006 mL, 0.063 mmol, 0.2 eq.) was added to give an orange solution. 4-(Prop-2-yn-1-yloxy)benzaldehyde (**S2**, 55.9 mg, 0.349 mmol, 1.1 eq.) was added (the colour of the solution changed to red) and the mixture was stirred under reflux until consumption of the indolinium **S1**, as monitored by TLC (12 h). The reaction mixture was allowed to cool down to rt, and the residue was purified by flash column chromatography using CH_2Cl_2 : MeOH (98 : 2) to afford (E)-1-ethyl-3,3-dimethyl-2-(4-(prop-2-yn-1-yloxy)styryl)-3H-indol-1-ium iodide (**3**, 90.0 mg, 0.197 mmol, 62.0%) as a brown solid, $\mathbf{R_f}$ = 0.5, MeOH : CH_2Cl_2 (5 : 95). **^{1}H NMR** (500 MHz, $CDCl_3$) δ 8.16 (d, *J* = 15.0 Hz, 1H), 7.98 (d, *J* = 7.9 Hz, 2H), 7.62 – 7.53 (m, 5H), 7.14 (d, *J* = 7.1 Hz, 2H), 4.82 (d, *J* = 2.4 Hz, 2H), 4.74 (s, 1H), 2.68 (s, 1H), 2.60 (t, *J* = 2.3 Hz, 1H), 1.82 (s, 5H). **^{13}C NMR** (126 MHz,

$CDCl_3$) δ 181.14 (C), 163.13(C), 155.22 (CH), 143.23 (C), 140.40 (C), 133.49 (CH), 129.90 (CH), 129.85 (CH), 127.50(C), 122.81(CH), 116.38 (CH), 114.52 (CH), 109.87 (CH),76.79 (CH), 56.36 (CH_2), 52.36 (C), 42.90 (CH_2), 27.19 (CH_3), 13.85 (CH_3). **LRMS** (*m/z, ESI*): 330.18 (M^+), 315.16, 276.14. **HRMS-ESI** Calculated for $C_{23}H_{24}NO$: 330.1852, found 330.1852.

(*E*)-1-ethyl-2-(4-hydroxystyryl)-3,3-dimethyl-3H-indol-1-ium iodide (**4**): Prepared using the abovementioned procedure using 1-ethyl-2,3,3-trimethyl-3*H*-indol-1-ium iodide (**S1,** 200 mg, 0.635 mmol, 1 eq.), piperidine (0.013 mL, 0.127 mmol, 0.2 eq.) and 4-hydroxybenzaldehyde **S3** (77.5 mg, 0.635 mmol, 1 eq.). The crude residue was purified by flash column chromatography using CH_2Cl_2 : MeOH (98 : 2 to 90 : 10 v/v) to afford (E)-1-ethyl-2-(4-hydroxystyryl)-3,3-dimethyl-3H-indol-1-ium iodide (**4**, 200.0 mg, 0.477 mmol, 75%) as a red solid. $\mathbf{R_f}$ = 0.59, MeOH:CH_2Cl_2 (10:90). **^{1}H NMR** (500 MHz, Methanol-*d*$_4$) δ 8.41 (d, *J* = 16.0 Hz, 1H), 8.02 – 7.97 (m, 2H), 7.78 – 7.72 (m, 2H), 7.65 – 7.57 (m, 2H), 7.43 (d, *J* = 16.1 Hz, 1H), 7.00 – 6.94 (m, 2H), 4.65 (q, *J* = 7.3 Hz, 2H), 1.83 (s, 6H), 1.56 (t, *J* = 7.3 Hz, 3H). **^{13}C NMR** (126 MHz, Methanol-*d*$_4$) δ 182.83 (C), 165.56 (C), 157.05 (CH), 144.85 (C), 141.86 (C), 134.76(CH), 130.49 (CH), 130.37 (CH), 127.43 (C), 124.03 (CH), 117.83 (CH), 115.38 (CH), 109.04 (CH), 53.49 (C), 42.92 (CH_2), 26.80 (CH_3), 13.77 (CH_3). **LRMS** (*m/z, ESI*): 292.17 (M^+), 277.15. **HRMS-ESI** Calculated for $C_{20}H_{22}NO$ (M^+): 292.1696, found 292.1695.

Procedure for the propargyl uncaging (exemplified for the uncaging of **1**)

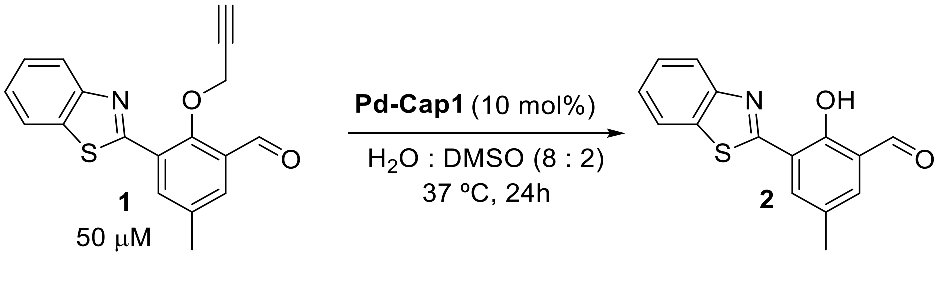

Under air, **1** (5 μL, 10 mM stock solution in DMSO, 1 eq.) was added to H_2O : DMSO mixture (8 : 2 v/v, 895 μL) at rt in a 1.5 mL HPLC vial with screw-cap. Then, **Pd-Cap1** (100 μL redispersed in water, 0.1 eq.) and a stirring bar were added, the vial sealed and the reaction mixture was stirred at 800 rpm at 37 ºC. At intervals of time (see Figure S4 below) the reaction mixture was centrifuged (8 minutes at 5000 rpm) to precipitate the **Pd-Cap1**. Aliquots (10 μL) of the crude mixture were diluted at 1 μM using H_2O : DMSO (3 : 7 v/v, 490 μL) and quantified using the fluorometer (See Fig S5-S6 for calibration curve).

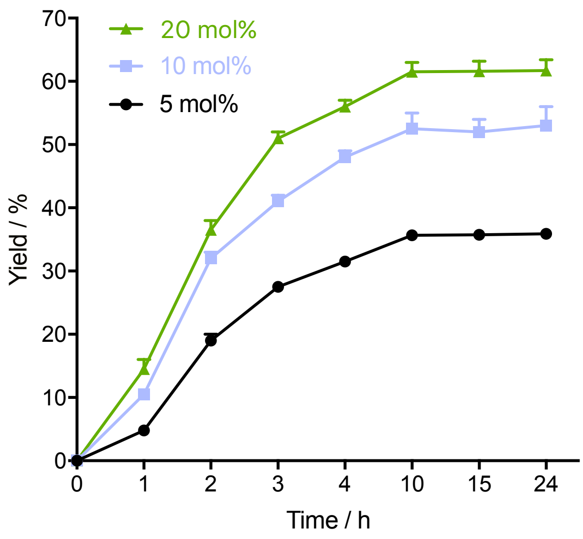

Figure S4. Time course of the reaction using different Pd concentrations (mean±s.e.m., *n* = 2).

a)

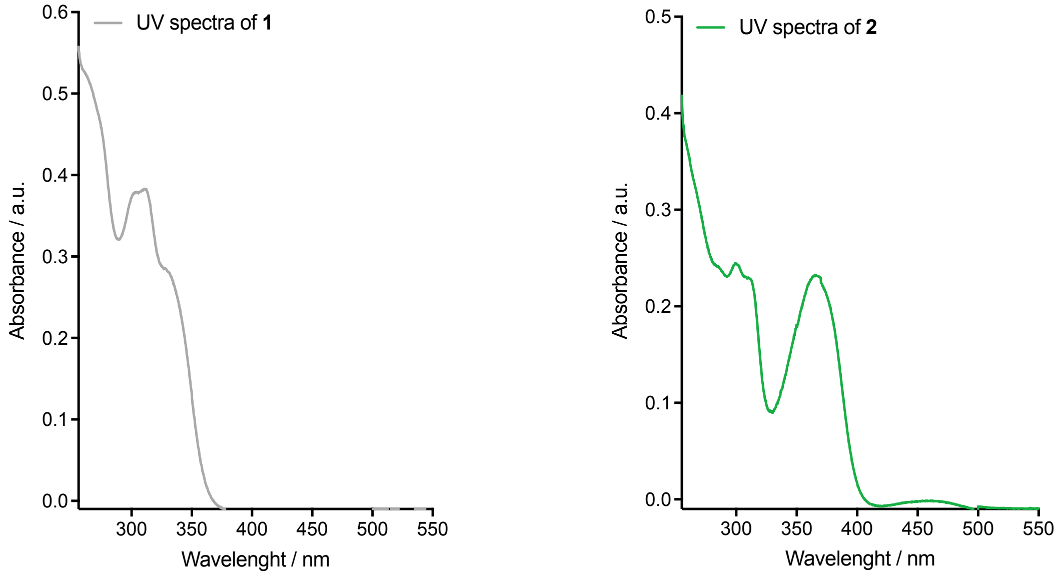

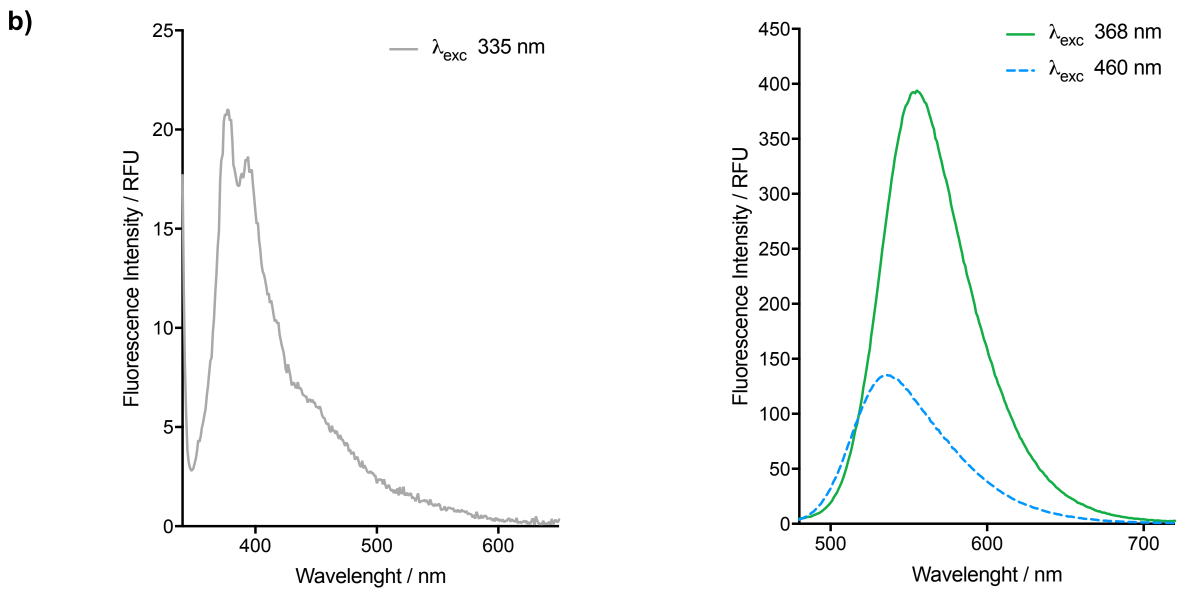

Figure S5. ***a)*** Absorption spectra of probe **1** (20 μM, 7 : 3 v/v mixture of DMSO : H_2O) (left) and **2** (20 μM, DMSO : H_2O 7 : 3) (right). ***b)*** Fluorescence spectra of **1** (5 μM, 7 : 3 v/v mixture of DMSO : H_2O) (left). Emission spectra of **2** (5 μM, 7 : 3 v/v mixture of DMSO : H_2O) at different wavelenght (λ_{ex} 368 nm, λ_{em} 558 nm, λ_{cut} 380 nm and λ_{ex} 460 nm, λ_{em} 535 nm, λ_{cut} 465 nm (right).

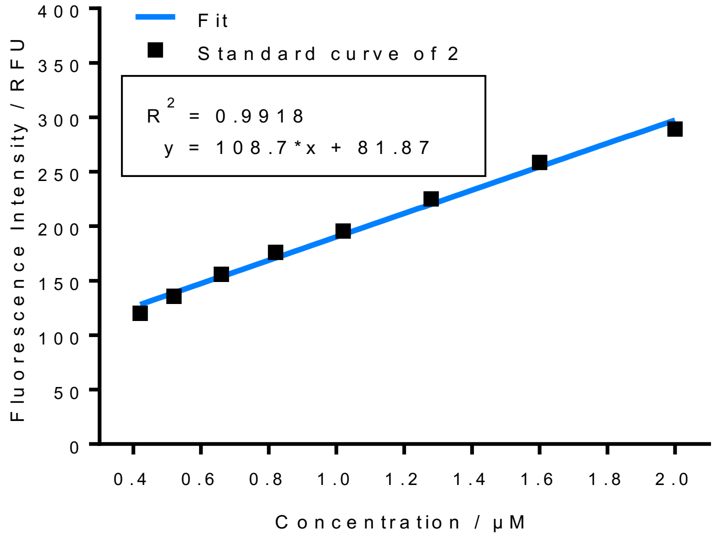

Figure S6. Calibration curve of **2**. Fluorescence intensity (λ_{ex} 460 nm, λ_{em} 535 nm, λ_{cut} 470 nm) at different concentrations in DMSO : H_2O (7 : 3).

(E)-1-ethyl-2-(4-hydroxystyryl)-3,3-dimethyl-3H-indol-1-ium iodide (**4**)

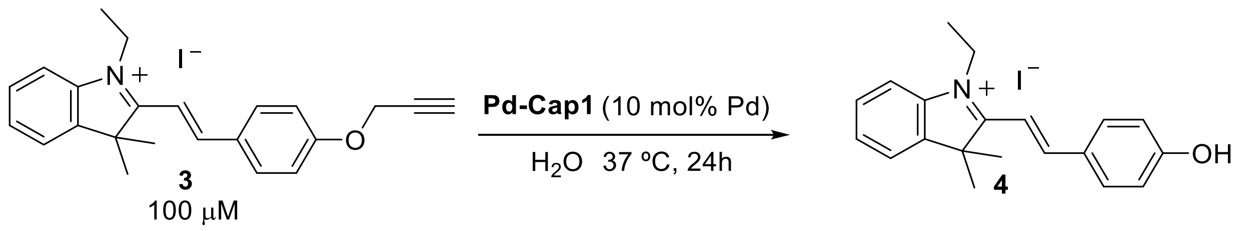

Using the general procedure for the uncaging of **1**, using a solution of **3** (10 μL, 10 mM stock solution in DMSO, 1 eq.), water (790 μL) and **Pd-Cap1** (200 μL redispersed in water, 0.1 eq.). After reaction completion, 500 μL of the crude mixture were diluted to 1 mL with water and analyzed by RP-HPLC-MS using coumarin as internal standard, yielding 47% of **4** (See Fig. S7 for RP-HPLC-MS chromatogram and calibration curve of **4**).

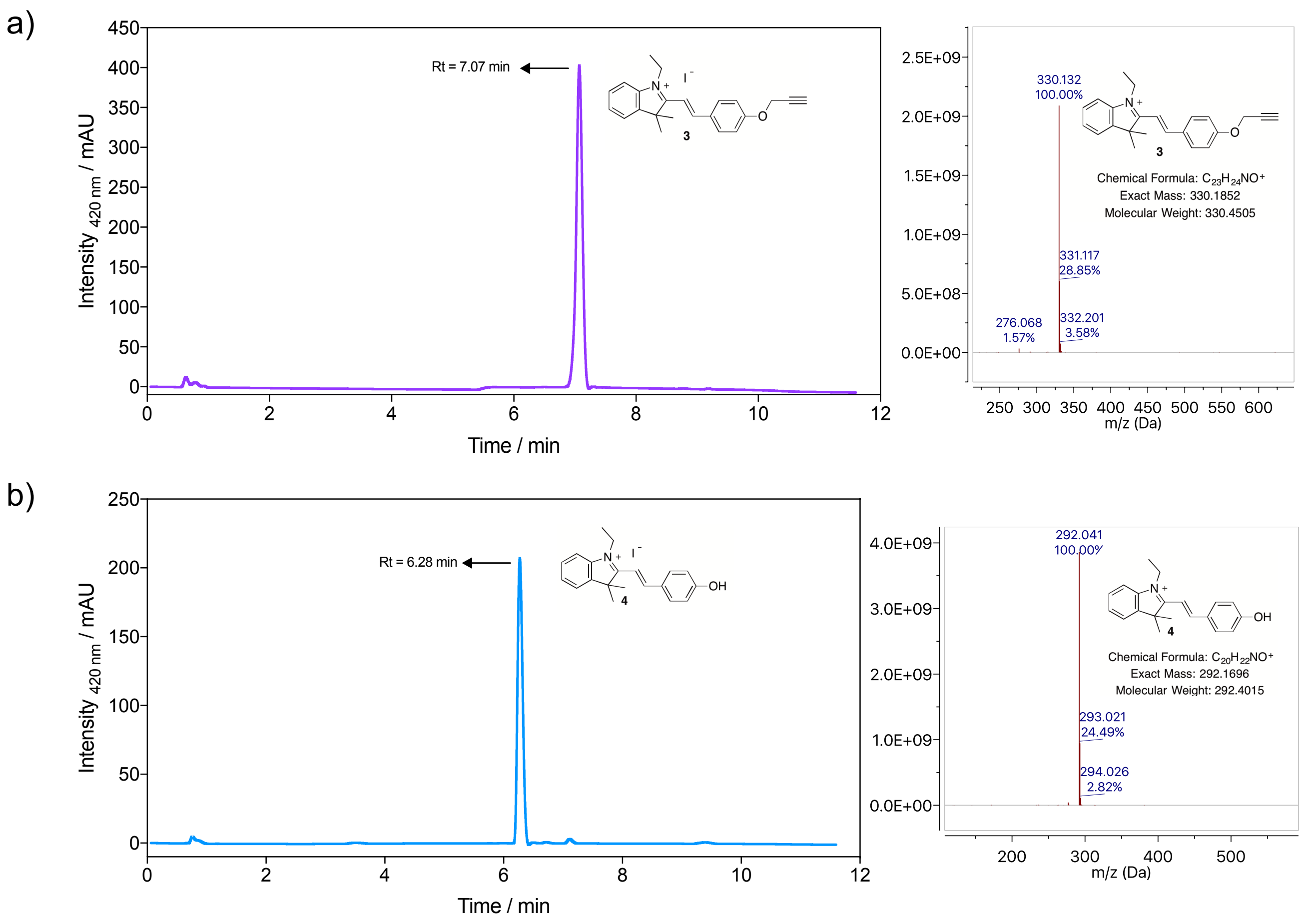

c)

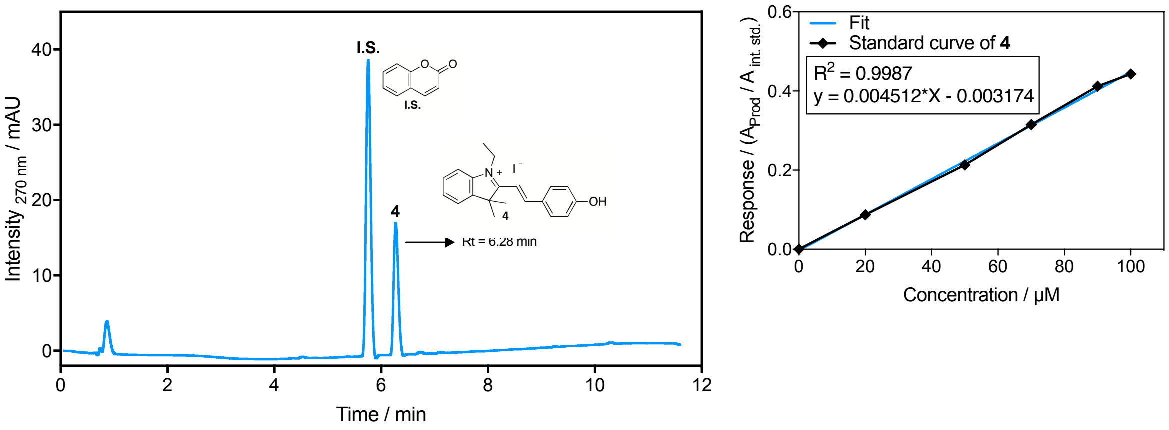

Figure S7. ***a)*** RP-HPLC-MS chromatogram (left) and ESI-MS spectra (right) of compund **3**, **b)** RP-HPLC-MS chromatogram (left) and ESI-MS spectra (right) of compund **4**; **c)** RP-HPLC-MS chromatogram (left) of compound **4** with coumarin (internal standard) and calibration curve (right).

General Procedure for the Suzuki-Miyaura cross coupling

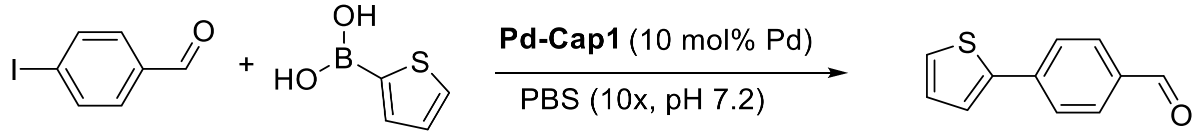

6 100 mM **5** 150 mM **7**

Under air, **6** (10 µL, 10 mM stock solution in DMSO, 1 eq.) was added to PBS (775 µL, 10X, pH = 7.2) at rt in a 1.5 mL HPLC vial with screw-cap. Then, boronic acid **5** (15 µL, 10 mM stock solution in DMSO, 1.5 eq.) was added followed by Pd-capsules **Pd-Cap1** (200 µL redispersed in water, 0.1 eq.) the vial sealed. A stirring bar was then added, the vial sealed and the reaction mixture was heated at 37 °C and stirred at 800 rpm. At intervals of time the crude mixture was centrifuged (8 minutes at 5000 rpm), aliquotes were taken, diluted to 10 µM (H_2O : EtOH, 1 : 1 v/v) and analyzed using Eclipse Fluorometer (λ_{ex} 330 nm, λ_{max} 434 nm, λ_{cut} 335 nm) yielding the product **7** in 86% yield after 24h. (See Fig. S8 for spectroscopic properties and calibration curve of **7**).

a)

b)

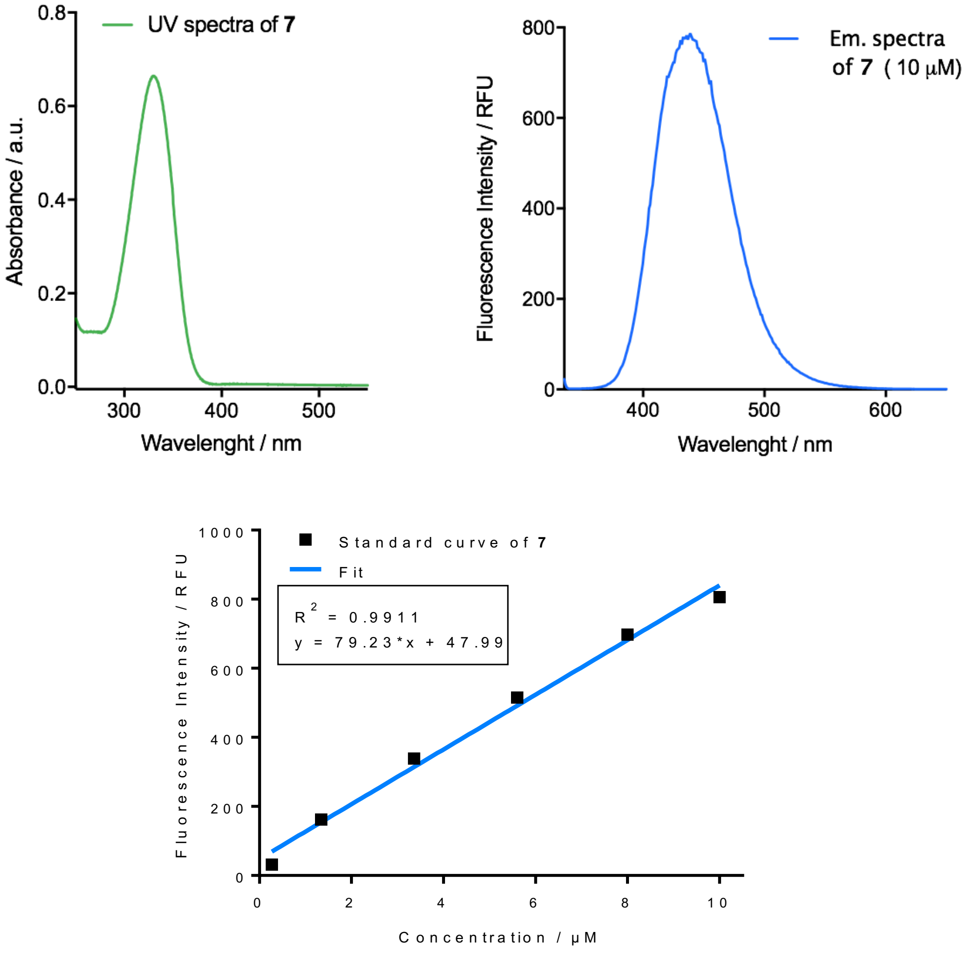

Figure S8. ***a)*** Absorption spectra (left) of **7** (20 μM in 1 : 1 v/v mixture of EtOH : H_2O) and fluorescence spectra (right) of **7** (10 μM in 1 : 1 v/v mixture of EtOH : H_2O, λ_{ex} 330 nm, λ_{em} 434 nm, λ_{cut} 335 nm). **b)** Calibration curve of **7**. Fluorescence intensity (λ_{ex} 330 nm, λ_{em} 434 nm, λ_{cut} 335 nm) at different concentrations in H_2O : EtOH (1:1).

To ensure that the observed fluorescent signal was generated only from the product, the following controls experiment have been done (Figure S9):

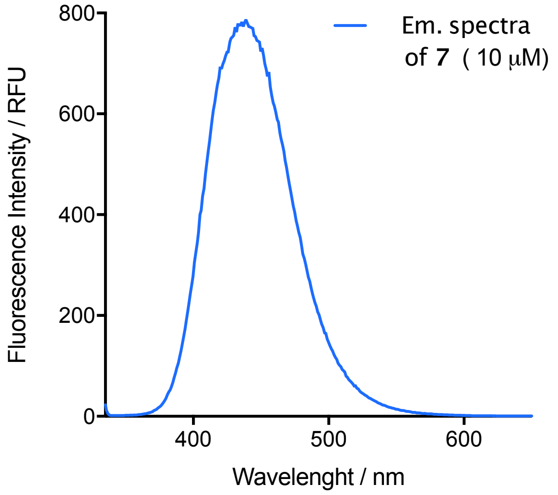

Fluorescence spectra of the product **7** (10 μM) (10 μM in 1 : 1 v/v mixture of EtOH : H_2O, λ_{ex} 330 nm, λ_{em} 434 nm, λ_{cut} 335 nm).

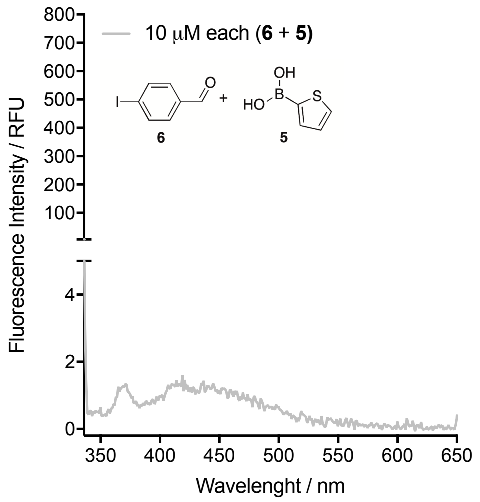

Fluorescence spectra of the mixture **5** and **6** (1 : 1 v/v mixture of EtOH : H_2O, λ_{ex} 330 nm, λ_{cut} 335 nm) in absence of **Pd-Cap1**.

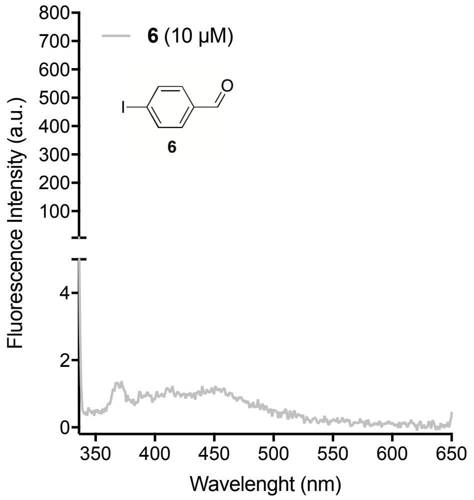

Fluorescence spectra of the reagent **5 (**1 : 1 v/v mixture of EtOH : H_2O, λ_{ex} 330 nm, λ_{cut} 335 nm) after 3h stirring with **Pd-Cap1** (10 mol%).

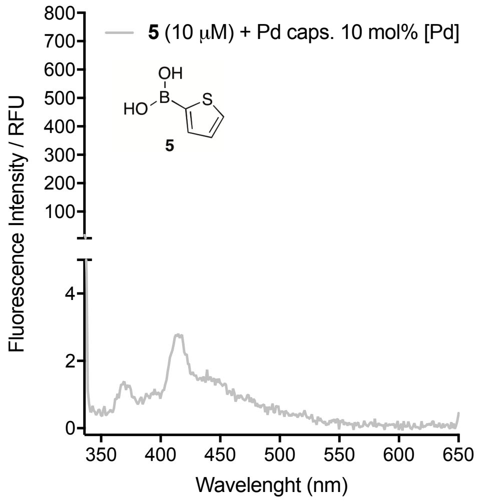

Fluorescence spectra of the reagent **6** (1 : 1 v/v mixture of EtOH : H_2O, λ_{ex} 330 nm, λ_{cut} 335 nm) after 3h stirring with **Pd-Cap1** (10 mol%).

Figure S9. Control mesurements

Time course of the Suzuki-Miyaura cross coupling:

Using the above mentioned general procedure for the Suzuki-Miyaura reaction, after 0.5 h, 2 h, 3 h, 5 h, 15 h and 24 h the reaction mixture was centrifuged, 25 μL of the supernatant were diluted to 10 μM with an EtOH : H_2O (1 : 1) mixture, and the conversion of reaction at

that particular time was quantified using calibration curve (Fig. S8). Results are included in Fig. S10.

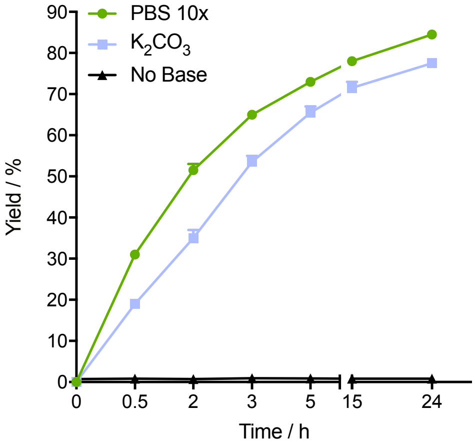

Figure S10. Reaction outcome in PBS (10X, pH = 7.2), with 10 eq. of K_2CO_3 and under base free conditions (mean±s.e.m., *n* = 2).

Procedures for catalysis in the presence of bio-additives

Suzuki-Miyaura cross coupling in the presence of additives (exemplified for the addition of glycine)

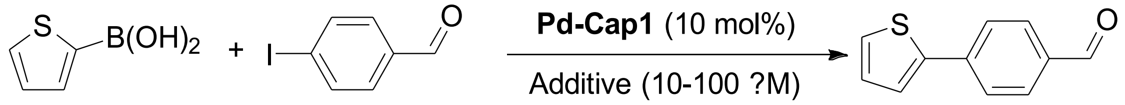

5 150 µM **6** 100 ?M **7**

Under air, **6** (2 µL, 50 mM stock solution in DMSO, 1 eq.) was added to water (794 µL) at room temperature followed by addition of boronic acid **5** (3 µL, 50 mM stock solution in DMSO, 1.5 eq.), glycine (1 µL, 10 mM stock solution in water, 0.1 eq.) and finally **Pd-Cap1** (200 µL redispersed in water, 0.1 eq.). A stirring bar was added, the vial sealed and the reaction mixture was stirred (800 rpm) at 37 ºC. After 3 h, the crude mixture was centrifuged (8 min at 5000 rpm) to precipitate the **Pd-Cap1**, 100 µL of the crude mixture were diluted to 1 mL with H_2O : EtOH (1 : 1) and quantified by fluorescence (see Fig. **S8b** for calibration curve).

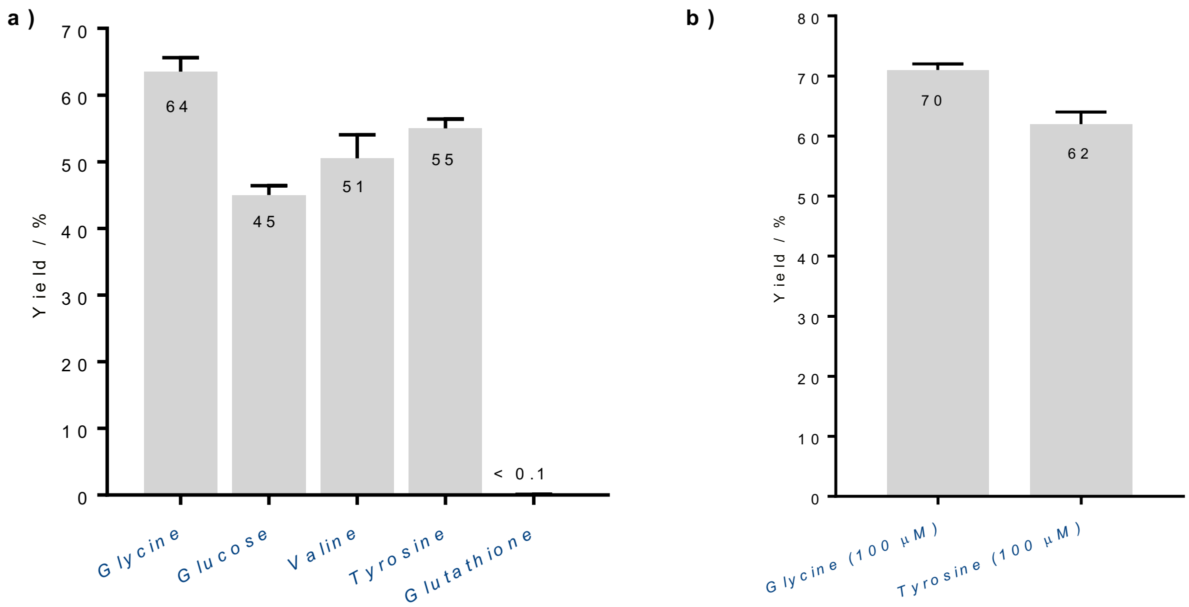

Figure S11. Pd-Cap1-promoted Suzuki-Miyaura reaction of **5** and **6** (top, mean ± s.e.m., n = 2) in presence of (**a**) 10 mol% of additives and (**b**) 1.0 equiv of additives

<u>Propargyl group uncaging in the presence of additives</u> (exemplified for the addition of glycine)

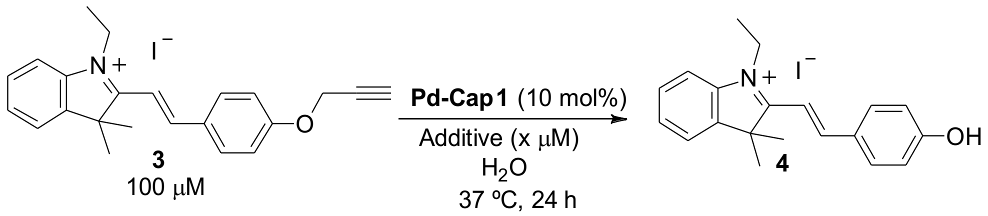

Under air, **3** (10 µL, 10 mM stock solution in DMSO, 1 eq.) was added to water (780 µL) at room temperature in a 1.5 mL HPLC vial with screw cap. Glycine (10 µL, 10 mM stock solution in water, 1 eq.) and **Pd-Cap1** (200 µL redispersed in water, 0.1 eq.) were added followed by a stirring bar, the vial sealed and the reaction mixture was stirred (800 rpm) at 37 ºC. After 24 h, the crude mixture was centrifuged (8 minutes at 5000 rpm) to precipitate the **Pd-Cap1**, 500 µL of the crude mixture were diluted to 1 mL with water and analyzed by RP-HPLC-MS using coumarin as internal standard.

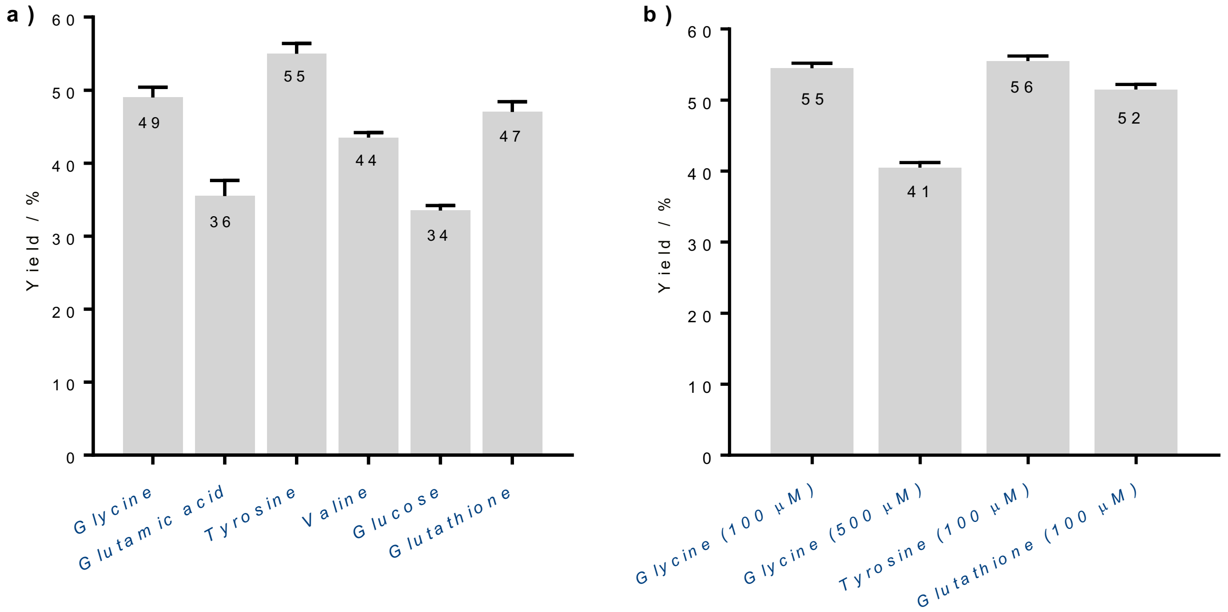

Figure S12*.* Pd-Cap1-promoted depropargylation reaction of **3** (top, mean ± s.e.m., n = 2) in presence of (**a**) 10 mol% of different additives and (**b**) 1.0-5.0 equiv of additives

Propargyl group uncaging under lysosomes-like conditions

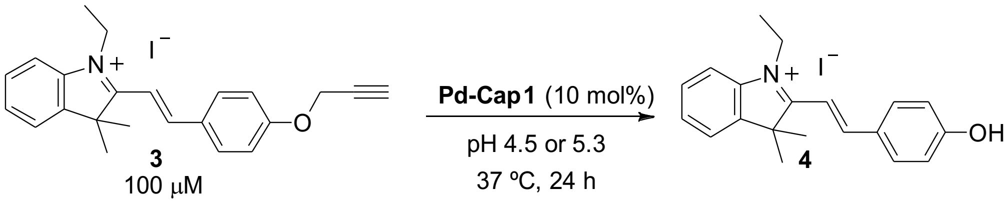

Under air, **3** (10 µL, 10 mM stock solution in DMSO, 1 eq.) was added to the buffer solution (790 µL) at room temperature in a 1.5 mL HPLC vial with screw cap followed by addition of **Pd-Cap1** (200 µL, redispersed in the same buffer solution, 0.1 eq.) and a stirring bar. The vial was sealed and the reaction mixture was stirred (800 rpm) at 37 ºC. After 24 h, the crude mixture was centrifuged (8 minutes at 5000 rpm) to precipitate the **Pd-Cap1**, 500 µL of the crude mixture were diluted to 1 mL with water and analyzed by RP-HPLC-MS using coumarin as internal standard.

Buffer solution	Yield (%)
CH_3CO_2H/CH_3CO_2Na (0.1 M), pH 4.5	36
CH_3CO_2Na buffer (3 M), pH 5.3	45

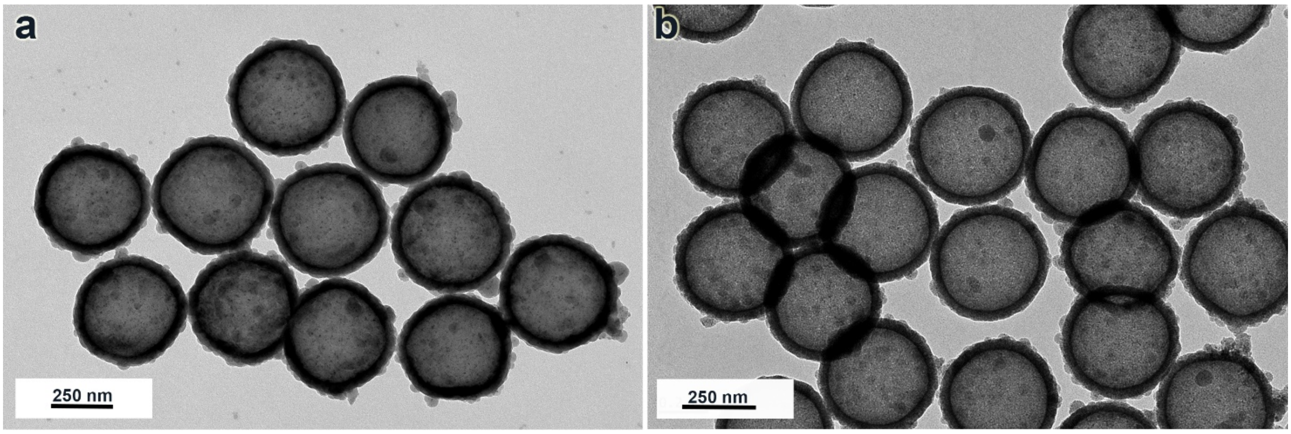

Figure S13. TEM images of **Pd-Cap1** before (a) and after (b) the performance of the abovementioned chemical reaction in simulated lysosomal conditions

Procedures for catalysis in cell cultures

Cell culture maintenance

All cell lines were cultured in DMEM (Dulbecco's modified Eagle's medium) supplemented with 10% (v/v) fetal bovine serum (FBS), 5 mM glutamine, penicillin (100 units/mL) and streptomycin (100 units/mL) (all from Invitrogen). Proliferating cell cultures were maintained in a 5% CO2 humidified incubator at 37 °C.

Cell viability assays

The toxicity of the different Pd-systems was tested by MTT assays in Vero cells as follows: 75000 cells per well were seeded in 96 well plates. 24 h later, cells were incubated with culture medium containing different concentrations of the catalysts for 24 h. Thiazolyl Blue Tetrazolium Bromide (Sigma) was then added to the cell culture medium to a final concentration of 0.5 mg/ml and incubated for 4 h to allow the formation of formazan precipitates by metabolically active cells. A detergent solution of 10% SDS (sodium dodecyl sulphate) and 0.01 M HCl was then added and the plate was incubated overnight at room temperature to allow the solubilization of the precipitates. The quantity of formazan in each well (directly proportional to the number of viable cells) was measured by recording changes in absorbance at 570 nm in a microtiter plate reading spectrophotometer (Tecan Infinite 200 PRO).

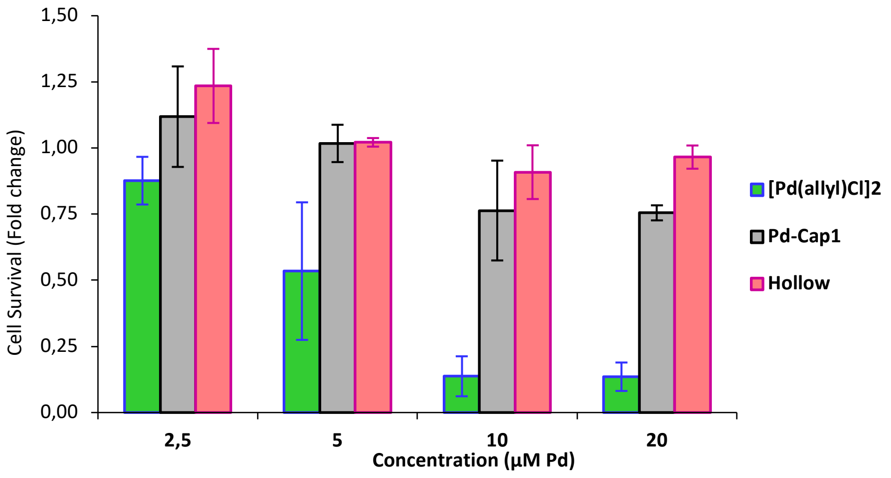

Figure S14. Viability of cells treated for 24 h with Pd-nanocapsules **Pd-Cap1** and the complex $[Pd(allyl)Cl]_2$. Hollow capsules not containing Pd were used as controls. Results are indicated as the fold change on cell viability with respect to untreated cells. Error bars represent the standard deviation of three independent experiments.

Depropargylation in cell culture

Vero cells were seeded on glass-bottom plates 24 h before treatment. Culture medium was removed, and cells were washed twice with serum-free DMEM. An incubation solution containing 5 μM Pd-Cap1 and 50 μM probe HBTPQ (**8**) in serum-free DMEM was added. After a 30-minute incubation, cells were washed twice with serum-free DMEM and observed in a confocal microscope with the following settings: λ_{ex}= 405 nm and a quadruple band emission filter.

Procedure to evaluate leaching from Pd-Capsules

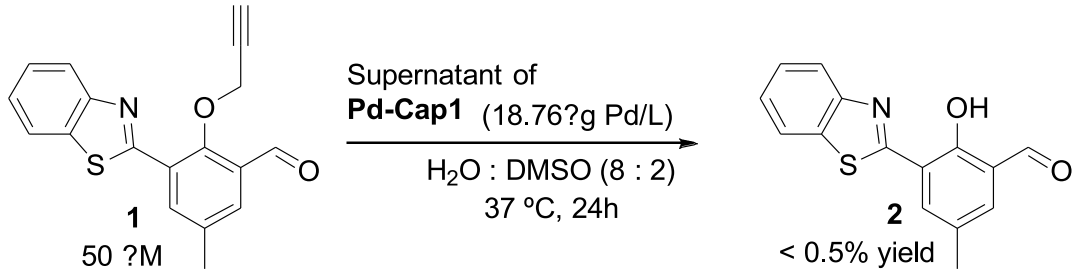

An aqueous solution (800 μL) of **Pd-Cap1** (200 μL, redispersed in water, [Pd] ≈ 10 μM) in a 1.5 mL HPLC vial with screw-cap was stirred at 37 ºC for 24 h. After that, the solution was centrifuged (8 min at 5000 rpm, ICP-AES of this supernatant showed a Pd content of 18.76 μg/L) and 500 μL of this solution were transferred in a new HPLC vial with screw-cap containing a solution of substrate **1** (5 μL, 10 mM stock solution in DMSO, 1 eq.) in H_2O : DMSO (500 μL, 1.5 : 1). The reaction mixture was stirred at 37 ºC for 24 h and the yield was determined by fluorescence using Eclipse Fluorometer (λ_{ex} 460 nm, λ_{em} 535 nm, λ_{cut} 470 nm) after centrifugation (8 min at 5000 rpm) and dilution to 2 μM (H_2O : DMSO 3 : 7), showing a yield below 0.5% (see Figure S15).

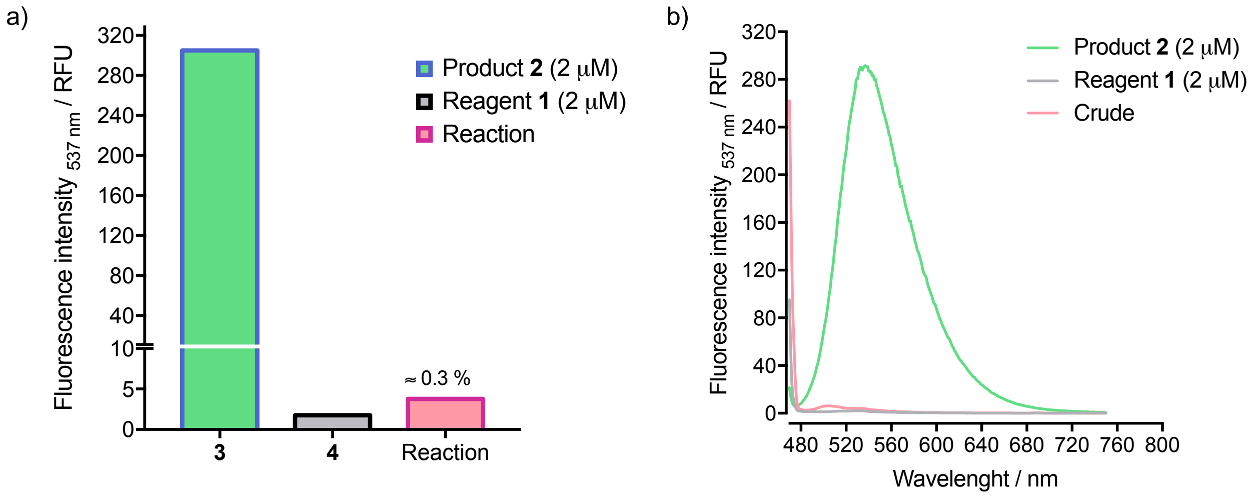

Figure S15. ***a)*** Fluorescence intensity of **3** (2 μM, left), **4** (2 μM, centre) and reaction mixture (2 μM, right) in H_2O : DMSO (3 : 7). The yield was determined using calibration curve of **4** (see Fig. S7c). **b)** Emission spectra of product **4**, reagent **3** and reaction crude in H_2O : DMSO (3 : 7). (λ_{ex} 460 nm, λ_{em} 535 nm, λ_{cut} 470 nm).

Recyclability test of Pd-Cap1

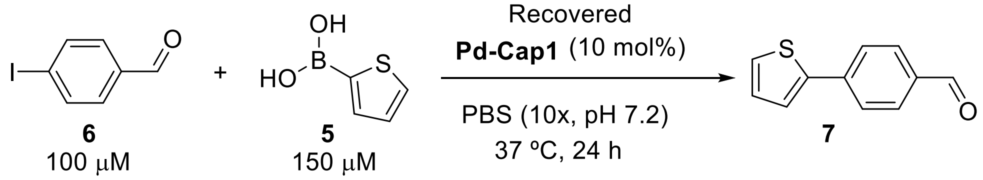

Pd-Cap1 were collected after Suzuki-Miyaura cross coupling reaction. **Pd-Cap1** were centrifuged at 8000 rpm for 10 minutes prior to quantification (the crude was diluted to 10 μM in H_2O : EtOH mixture 1 : 1 and analyzed with Eclipse Fluorometer, λ_{ex} 330 nm, λ_{max} 434 nm, λ_{cut} 335 nm), washed with EtOH and centrifuged as above (the procedure was repeated 3 times). The solid residue of **Pd-Cap1** was diluted with PBS (10X, pH = 7.2) and used for other 2 experiments following the general procedure abovementioned described.

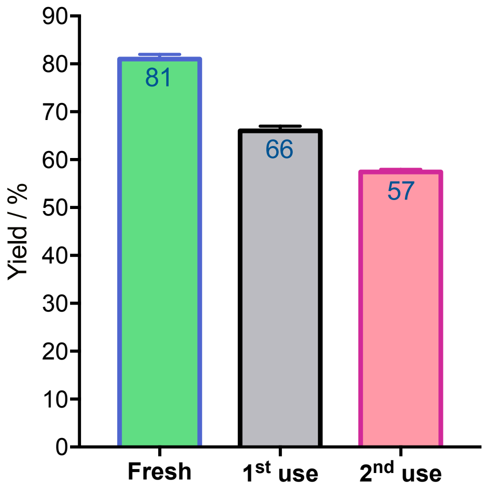

Figure S16. Recyclability of **Pd-Cap1** for the Suzuki-Miyaura cross coupling reaction (mean ± s.e.m., n = 2).

REFERENCES

1. J. S. Lindsey, P. A. Brown, and D. A. Siesel,*Tetrahedron*, 1989, **45**, 4845.
2. Y. Kumar, A. Matta, P. Kumar, V. S. Parmar, E. V. Van der Eycken and B. K. Singh, *RSC Adv*., 2015, **5**, 1628.
3. T. Chen, T. Wei, Z. Zhang, Y. Chen, J. Qiang, F. Wang, and X. Chen, *Dyes and Pigments*, 2017, **140**, 392.
4. K. Guo, Z. Gao, J. Cheng, Y. Shao, X. Lu and H. Wang, *Dyes and Pigments*, 2015, **115**, 166.
5. T. Gao, P. Xu, M. Liu, A. Bi, P. Hu, B. Ye, W. Wang and W. Zeng, W. *Chem. Asian J.* 2015, **10**, 1142.
6. B. Lim, M. Jiang, P. H. C. Camargo, Y. Zhu and Y. Xia, *Adv. Funct. Mater*, 2009, **19**, 189.

NMR Spectra

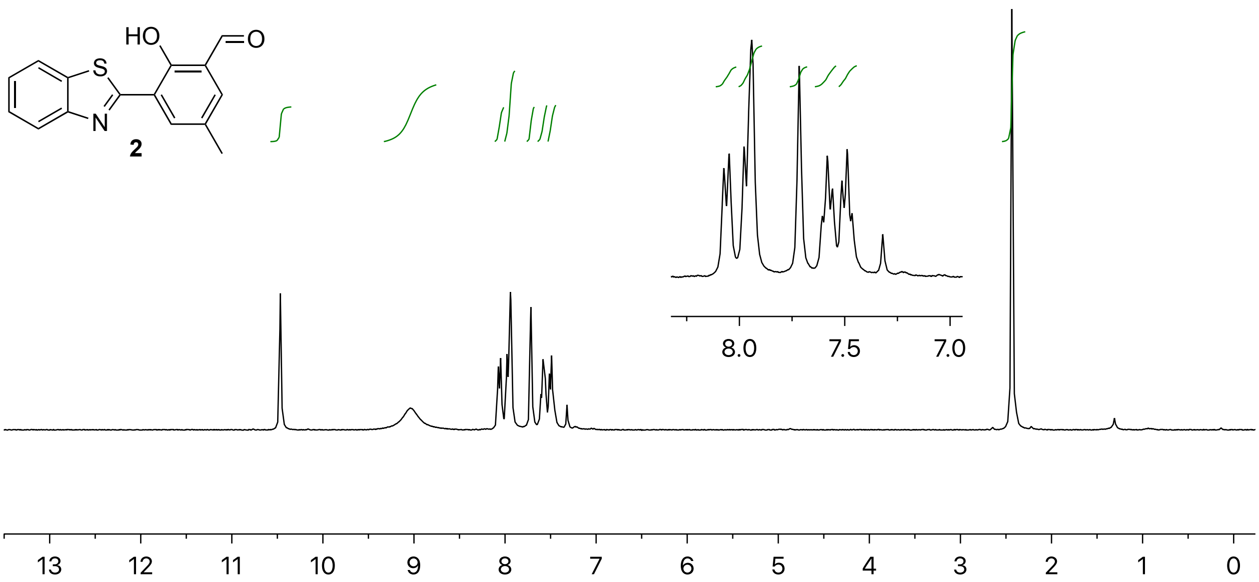
HO
O
S
N
2
8.0
7.5
7.0
13
12
11
10
9
8
7
6
5
4
3
2
1
0

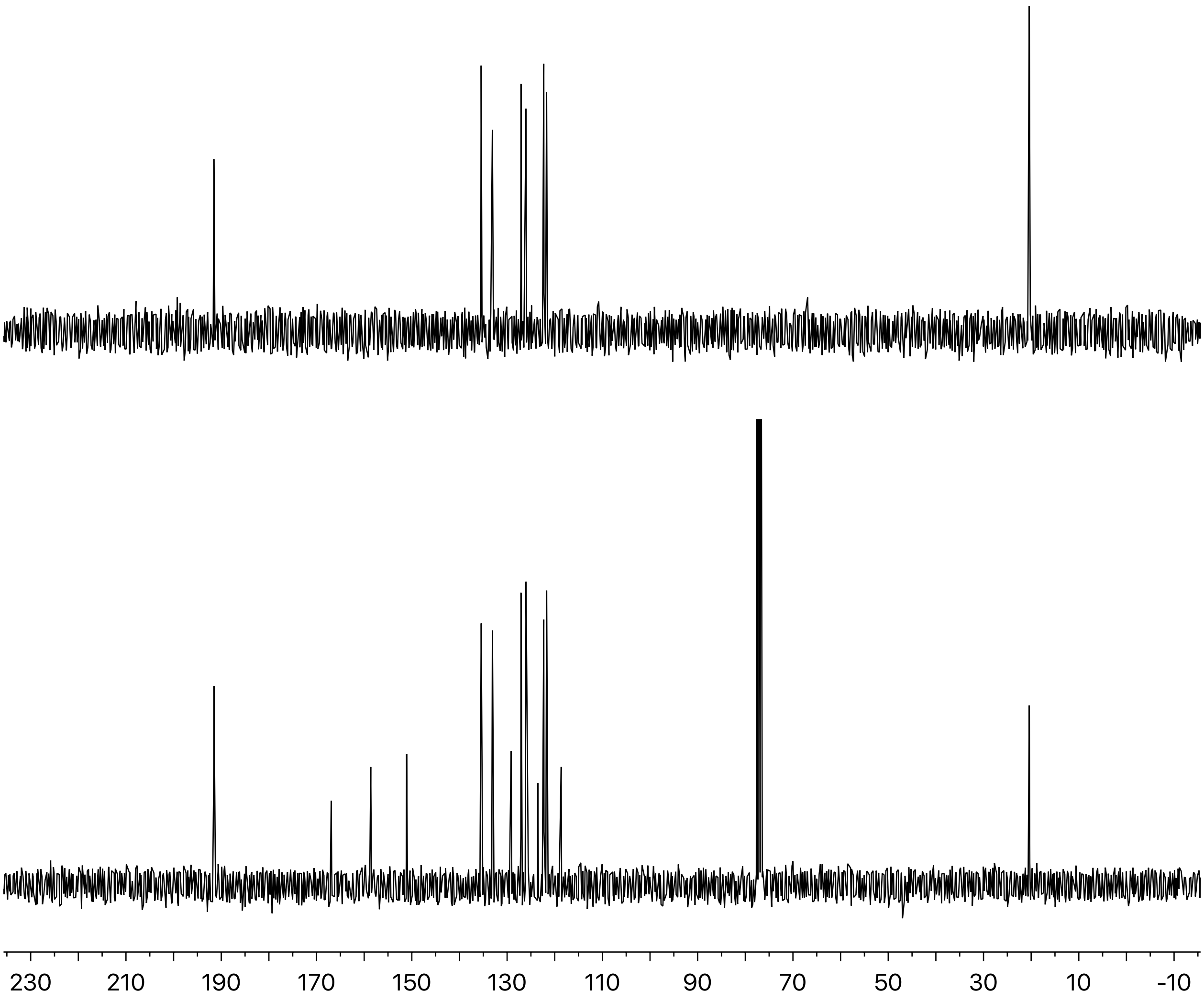
230
210
190
170
150
130
110
90
70
50
30
10
-10

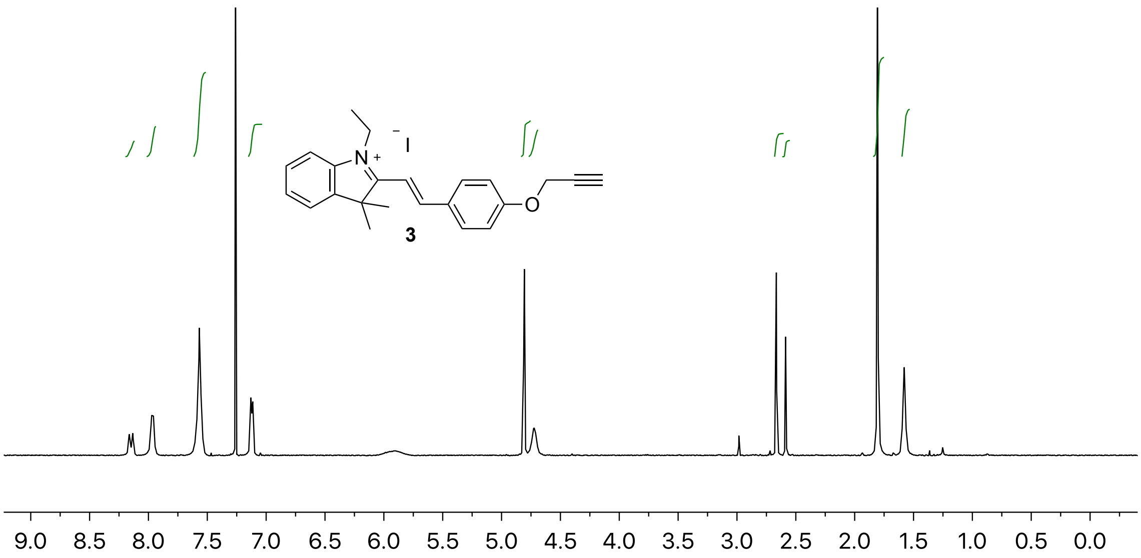
N+
I
O
3
9.0 8.5 8.0 7.5 7.0 6.5 6.0 5.5 5.0 4.5 4.0 3.5 3.0 2.5 2.0 1.5 1.0 0.5 0.0

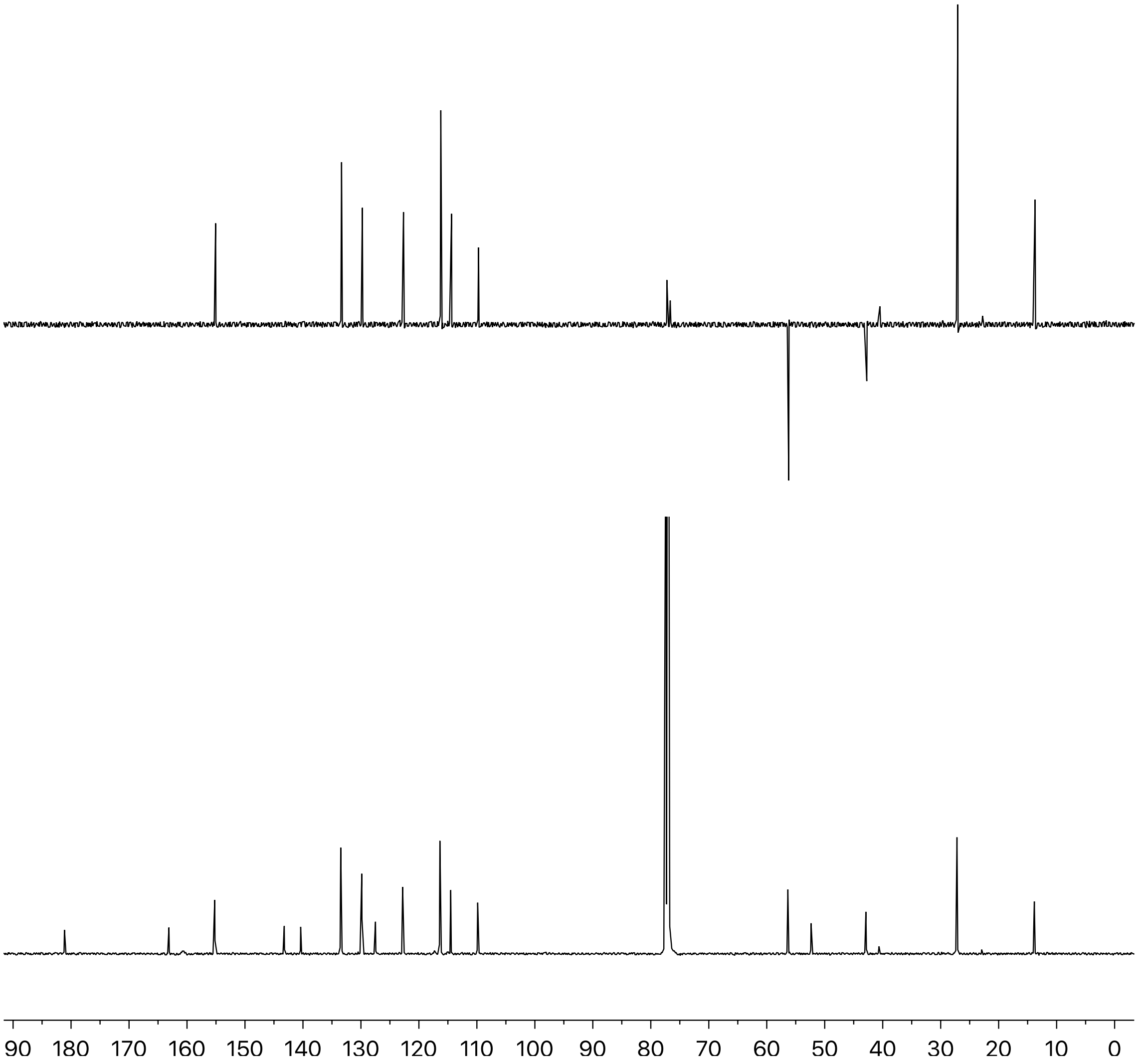
90 180 170 160 150 140 130 120 110 100 90 80 70 60 50 40 30 20 10 0

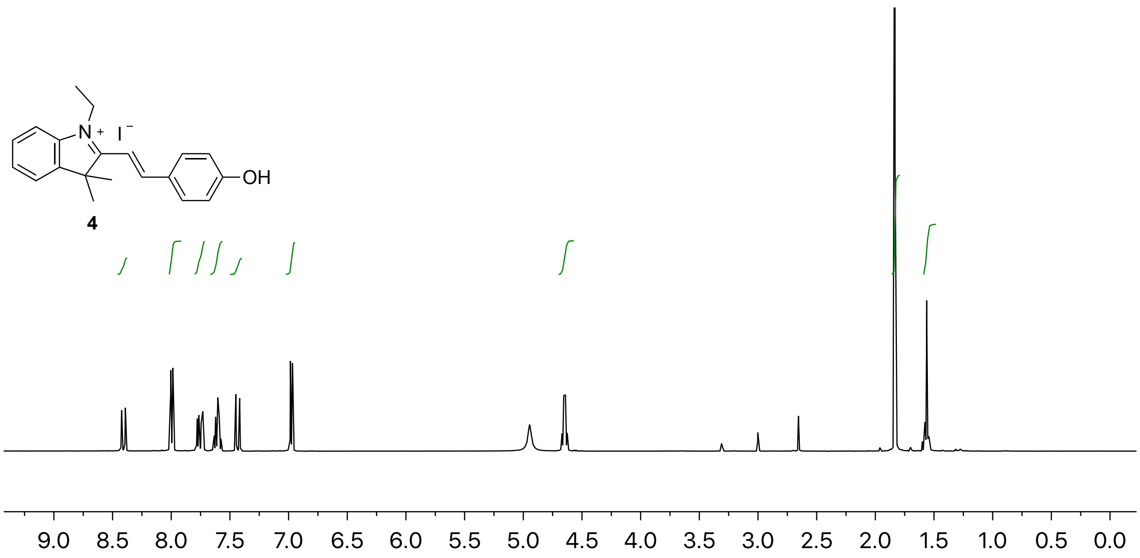
N+
I−
OH

4

9.0 8.5 8.0 7.5 7.0 6.5 6.0 5.5 5.0 4.5 4.0 3.5 3.0 2.5 2.0 1.5 1.0 0.5 0.0

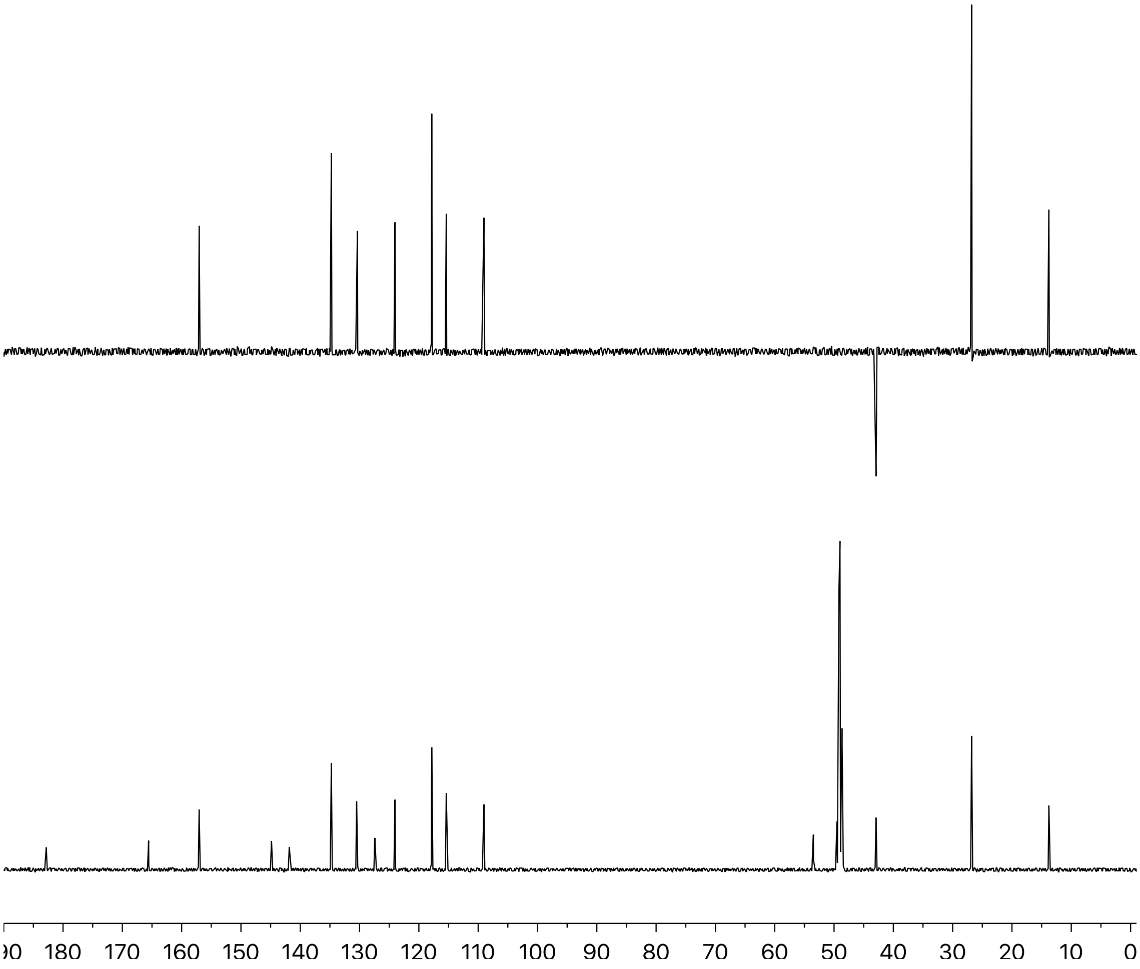
180 170 160 150 140 130 120 110 100 90 80 70 60 50 40 30 20 10 0

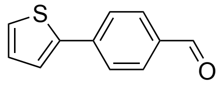
S
O

7

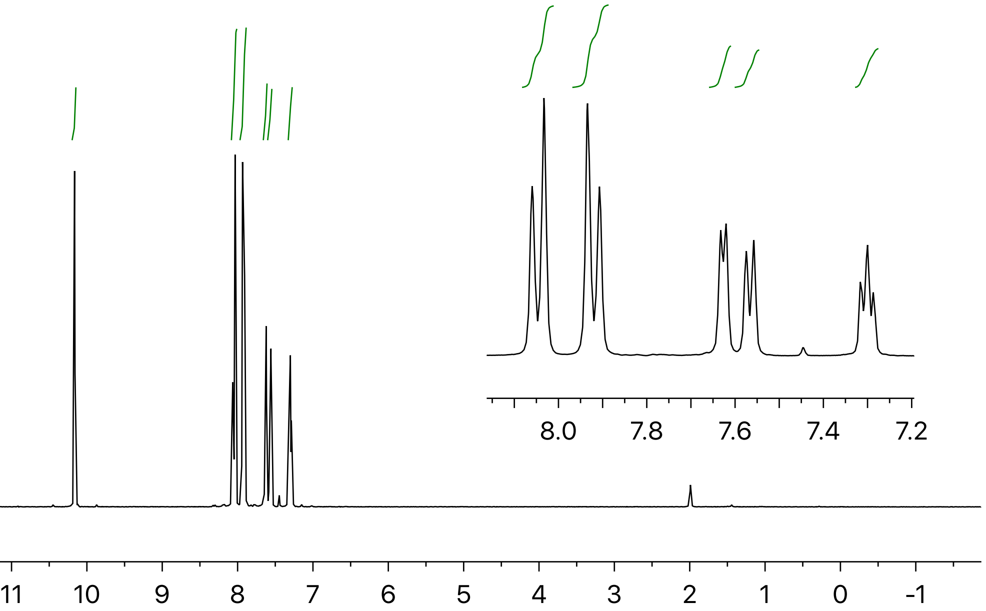
8.0
7.8
7.6
7.4
7.2
15
14
13
12
11
10
9
8
7
6
5
4
3
2
1
0
-1

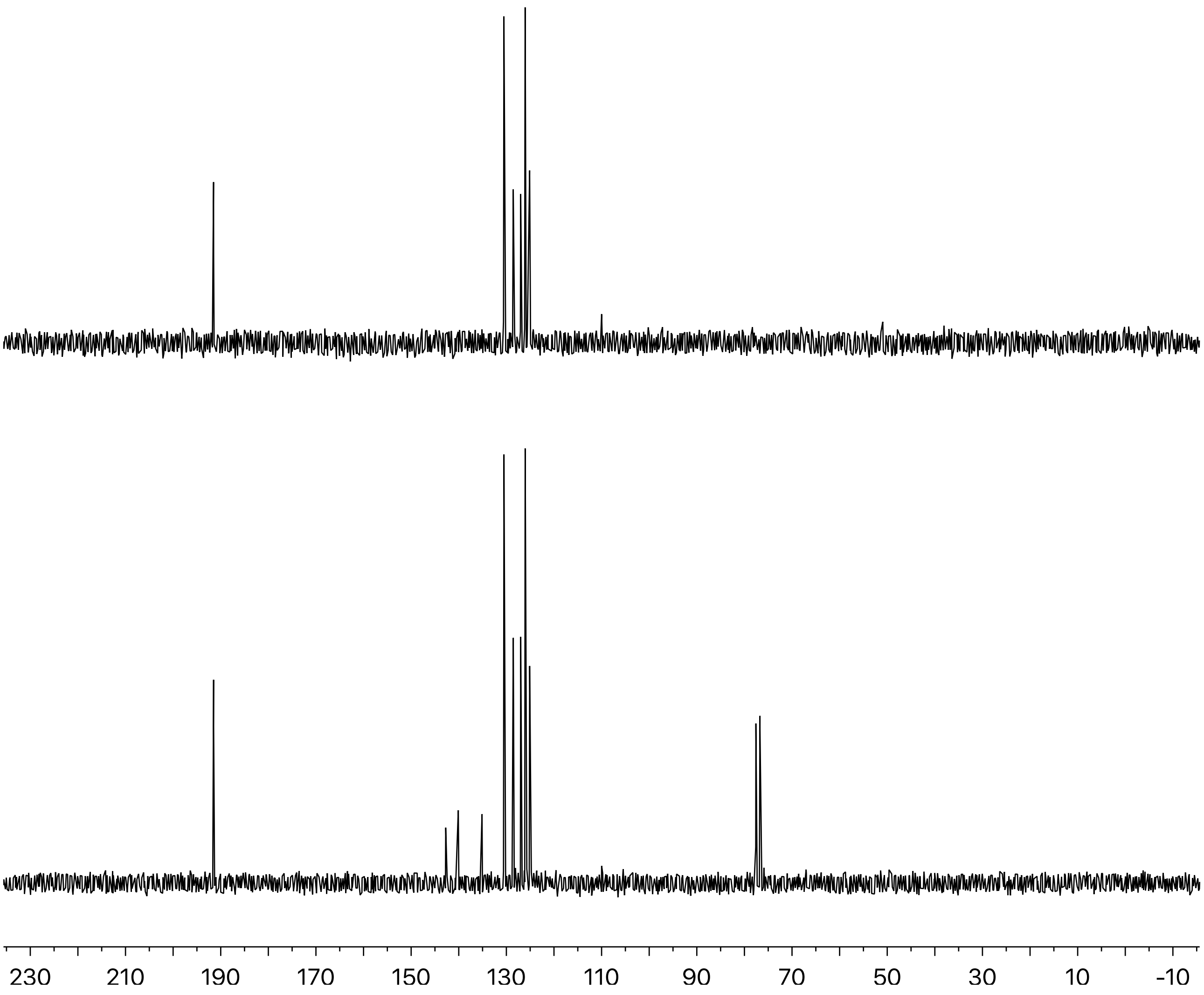
230
210
190
170
150
130
110
90
70
50
30
10
-10
