# Supplementary material for: Hollow nanoreactors for Pd-catalyzed Suzuki–Miyaura coupling and O-propargyl cleavage reactions in bio-relevant aqueous media
Source: Chem Sci. 2018 Dec 26;10(9):2598–603. doi: 10.1039/c8sc04390f (PMC6419927; doi:10.1039/c8sc04390f)
Supplement: Supplementary file 2 [file SC-010-C8SC04390F-s002.pdf]

Electronic Supplementary Material (ESI) for Chemical Science.
This journal is © The Royal Society of Chemistry 2019

Quest for biocompatible nanoreactors reaches a new milestone

Palladium (Pd) metal nanoparticles (NP) are good catalysts that can mimic enzyme activity in living systems...

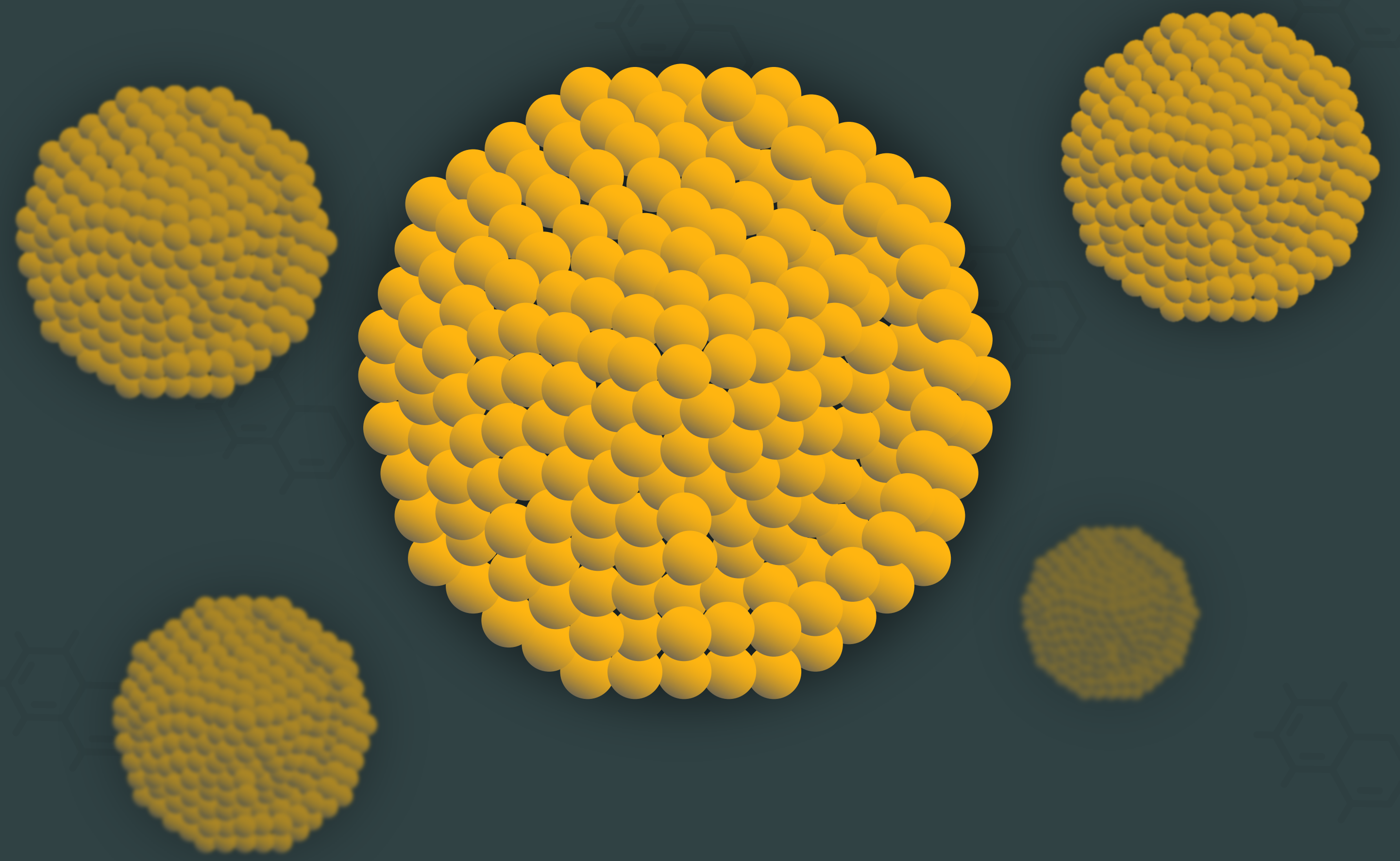

but most nanocatalysts only work in organic solvents, and become aggregated and deactivated in aqueous solvents.

Encapsulating Pd NP could overcome these limitations

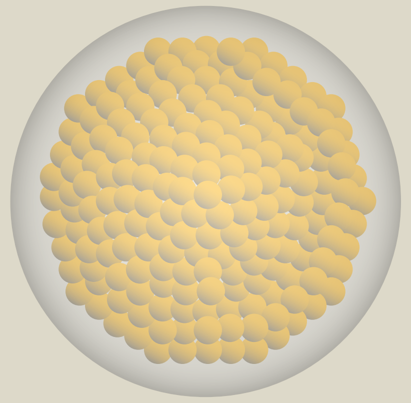

Synthesis of hollow spheres with Pd-NP at their inner cavity

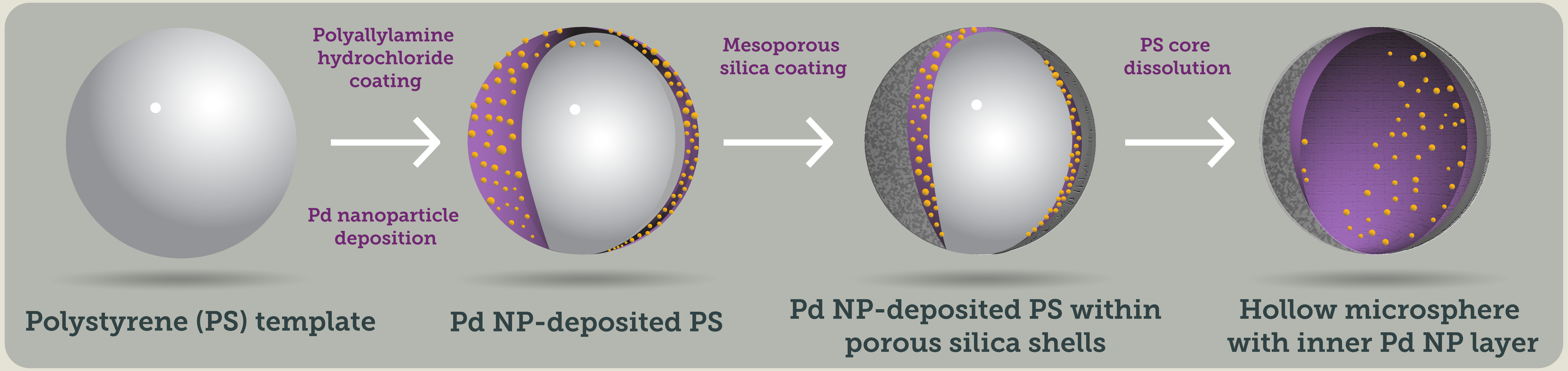

How are hollow-shelled Pd nanoreactors better than free NP?

Biocompatible

Stable at 37 °C

Prevents entry of biomolecules and leakage of Pd

Stable in aqueous solutions

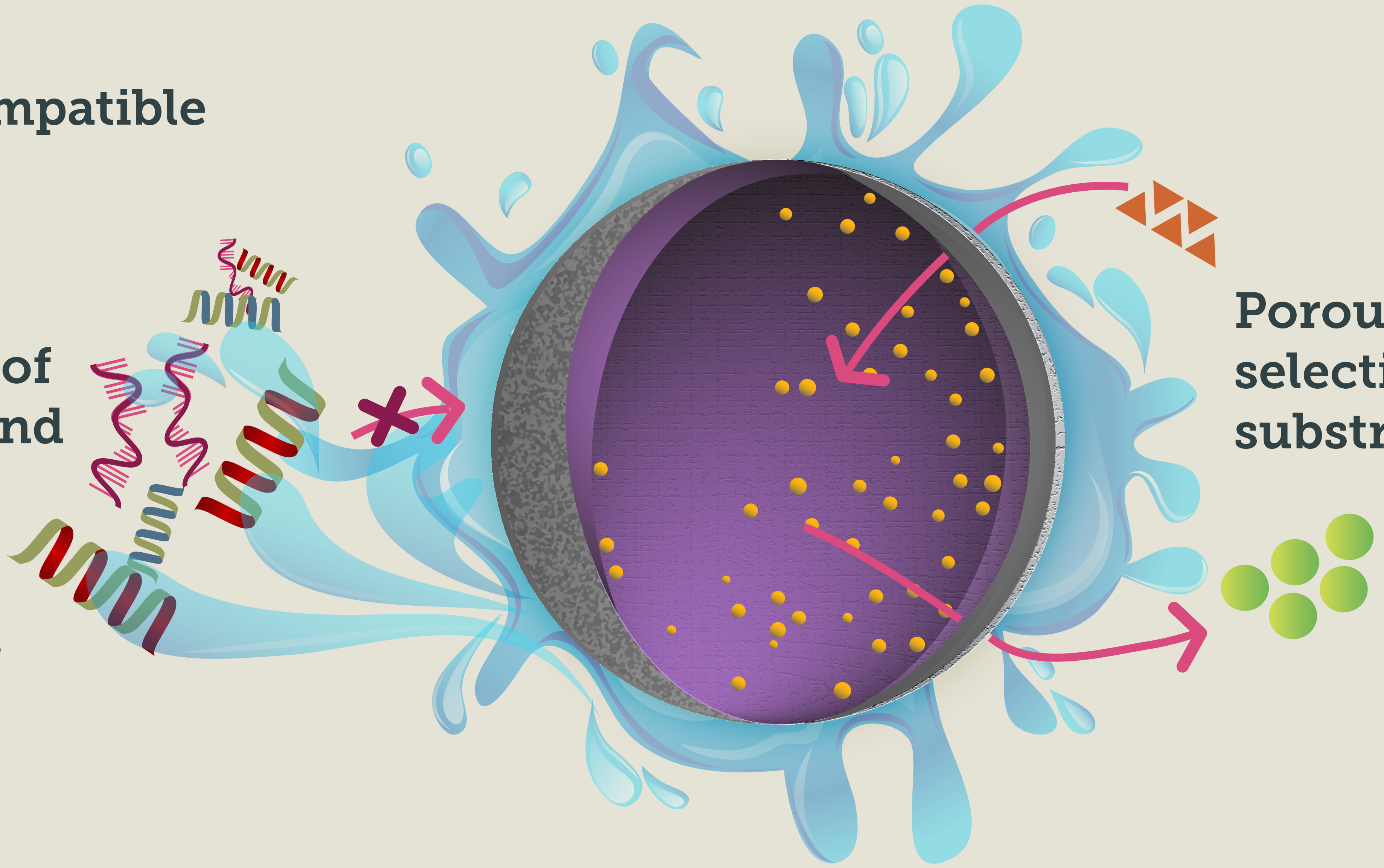

Low biotoxicity

Porous surface enables selective passage of substrates and products

Easy recovery by centrifugation

Novel biocompatible hollow-shelled Pd nanoreactors can be used for delivery of targeted therapies

Chemical Science

PICK OF THE WEEK

Hollow nanoreactors for Pd-catalyzed Suzuki–Miyaura couplings and *O*-propargyl cleavage reactions in bio-relevant aqueous media

López, Correa-Duarte, Mascareñas *et al.* (2019) | DOI: 10.1039/c8sc04390f

ROYAL SOCIETY OF CHEMISTRY
